# Proteasome activator Blm10 levels and autophagic degradation directly impact the proteasome landscape

**DOI:** 10.1016/j.jbc.2021.100468

**Published:** 2021-02-25

**Authors:** Alicia Burris, Kenrick A. Waite, Zachary Reuter, Samuel Ockerhausen, Jeroen Roelofs

**Affiliations:** 1 Department of Biochemistry and Molecular Biology, University of Kansas Medical Center, Kansas City, Kansas, USA; 2 Molecular, Cellular, and Developmental Biology Program, Division of Biology, Kansas State University, Manhattan, Kansas, USA

**Keywords:** proteasome, protein degradation, autophagy, yeast, ubiquitin, proteasome storage granule, stress response, Blm10, CP, core particle, PSG, proteasome storage granule, RP, regulatory particle, YPD, yeast peptone dextrose

## Abstract

The proteasome selectively degrades proteins. It consists of a core particle (CP), which contains proteolytic active sites that can associate with different regulators to form various complexes. How these different complexes are regulated and affected by changing physiological conditions, however, remains poorly understood. In this study, we focused on the activator Blm10 and the regulatory particle (RP). In yeast, increased expression of Blm10 outcompeted RP for CP binding, which suggests that controlling the cellular levels of Blm10 can affect the relative amounts of RP-bound CP. While strong overexpression of *BLM10* almost eliminated the presence of RP-CP complexes, the phenotypes this should induce were not observed. Our results show this was due to the induction of Blm10-CP autophagy under prolonged growth in YPD. Similarly, under conditions of endogenous *BLM10* expression, Blm10 was degraded through autophagy as well. This suggests that reducing the levels of Blm10 allows for more CP-binding surfaces and the formation of RP-CP complexes under nutrient stress. This work provides important insights into maintaining the proteasome landscape and how protein expression levels affect proteasome function.

Most protein degradation in eukaryotic cells is performed by a large complex known as the proteasome. Unlike lysosomal and secreted proteases, proteasomes sequester proteolytic active sites away from potential substrates as they are located within a barrel-shaped structure known as the core particle (also known as CP or 20S). CP is composed of two sets of 14 unique subunits. These subunits are arranged in four stacked heptameric rings. α subunits 1–7 form the outer rings while the two inner rings are composed of β subunits 1–7. Three pairs of proteolytic β subunits, each with distinct specificity, are responsible for cleaving substrates into short polypeptides (1, 2). Peptides released by the proteasome are further processed by cytosolic peptidases to produce intermediates for various metabolic processes (3, 4).

While the CP alone appears to be able to degrade certain classes of substrates, *e.g.*, under oxidative stress, it is well established that the majority of substrates cannot be degraded by CP alone (5). The reason for this is threefold; first, most substrates are labeled for degradation with a posttranslational modification on lysine residues known as ubiquitination. The receptors that recognize this modification are not part of the CP. Second, most substrates contain stable tertiary and quaternary structures, which require unfolding before they can enter the CP. Third, even when substrates are unfolded or disordered, entry into the catalytic chamber is restricted by a gate composed of the N termini of α subunits (5).

The association of CP with the regulatory particle (RP or 19S) eliminates all of these limitations and results in the formation of the 26S proteasome (here used to refer to CP complexes with one or two RPs). The RP is composed of 19 polypeptides. Three of these function as intrinsic ubiquitin receptors, Rpn1, Rpn10, and Rpn13, and thus are able to bind substrates (6, 7, 8, 9). These substrates are deubiquitinated by various deubiquitinating enzymes including the intrinsic RP subunit Rpn11 and the proteasome-associated enzyme Ubp6. A hexameric ring of six AAA-ATPases (Rpt1-6) utilizes ATP to unfold substrates and translocate them into the CP for degradation. Several of the ATPase subunits have C-terminal tails with a conserved Hb-Y-X motif (Hb refers to a hydrophobic amino acid, Y is tyrosine, and X can be any amino acid). Docking of these tails into pockets on the surface of the CP α-ring contributes to the affinity between RP and CP as well as induces conformational changes that open the gate and allow for substrate entry into the core particle (10, 11, 12). Thus, 26S proteasomes bind, unfold, and subsequently degrade the majority of physiologically important substrates.

Besides RP, several other complexes can associate with the same surface of CP that is occupied by RP, namely the 11S activator (REGα-β and REGγ, a.k.a. as PA28αβ and PA28γ; not found in yeast), Pba1-Pba2/PAC1-PAC2, Blm10/PA200, and Fub1/PI31 (11, 13, 14, 15). However, none of these can hydrolyze ATP or are able to recognize ubiquitinated substrates suggesting that their function is either different or more specialized. In general terms, they could function as a competitor to prevent RP binding. Competitive binding has the potential to negatively regulate proteasome activity, as has been found and proposed for PI31 (15, 16). A second possible function involves a role during CP assembly or maturation. Pba1-Pba2 was shown to stimulate α-ring assembly and prevented RP from associating with immature CP (17, 18). Another possible function is in regulating the localization or transport of CP, *e.g.*, into or out of the nucleus. Such a role has been proposed for Blm10 in yeast (19). Finally, these CP-associated proteins may function as distinct, specialized degradation complexes. Here, the degradation would not depend on ubiquitination-based substrate targeting (considering the lack of ubiquitin receptors) or protein unfolding (considering the lack of ATPase activity in these regulators). This role is consistent with the proposed function for REGγ in degrading intrinsically disordered proteins in the nucleus (20, 21, 22, 23, 24).

The above models act under the assumption that CP forms homogeneous complexes where one type of activator binds both ends of the CP; however, CP can actually form hybrid complexes where RP binds to one end, while the other end is occupied by one of these alternative regulators. Such complexes have been purified from cells and detected in cell lysates (25, 26, 27). The formation of hybrid complexes could cause a change in cellular localization of RP-CP complexes. Binding of other activators may also induce allosteric changes inside the CP that affect the specificity of proteolytic cleavage or change cleavage dynamics and peptide retention in the catalytic chamber. As such, hybrid complexes could degrade ubiquitinated proteins, but produce peptides of different composition and length as final products that are released by the proteasome. REGα-β forms heptameric rings that stimulate gate opening through a mechanism different from RP. The C-terminal Hb-Y-X motifs of REGα-β contribute to CP binding but are not involved in gate opening. Instead, gate opening occurs through an activation loop within REGα-β that induces conformational changes required for access (28, 29).

Blm10 is a unique regulator as it has been observed bound to both mature and immature CP. Blm10 can be bound to CP alone or in hybrid complexes with RP. Blm10 in *Saccharomyces cerevisiae* (PA200 in humans) is a single ∼240 kDa polypeptide that contains multiple heat repeats, a bromodomain-like region, and an Hb-Y-X motif at its C terminus. Similar to other activators, this motif allows Blm10 to bind to a pocket on the surface of the α ring, specifically the α5-α6 pocket (11). However, unlike other regulators, Blm10 binds proteasomes as a monomeric protein. Binding of Blm10 to the CP induces partial gate opening and increases peptidase activity; therefore, Blm10 has been described as an activator (30). Indeed, Blm10-CP complexes have been reported to be involved in degradation of short peptides and unstructured proteins such as tau, which is reasonable considering those do not require ubiquitination or ATPase-dependent unfolding (30). In addition, Blm10 appears to be required for the degradation of Sfp1 and histones, further supporting a role for Blm10 in protein degradation (31, 32, 33). The presumably folded nature of these substrates might indicate a role for hybrid RP-CP-Blm10 complexes. However, a clear mechanism of action for these hybrid complexes is unknown and other factors might assist in the degradation.

Considering that Blm10 is also found on an immature form of CP, a role in CP maturation has also been proposed. However, its function here seems very different from the assembly chaperones Pba1-Pba2, which seems to exclusively bind to immature CP. Pba1-Pba 2 has a very low affinity for mature CP and a high affinity for the immature form (18). Consistent with this, Pba1-Pba2 is more embedded in the α ring of immature CP compared with mature CP, thus restricting RP-CP interactions (34). While Blm10 could potentially perform a similar role for immature CP, there is no apparent difference in Blm10 affinity for mature *versus* immature CP that would allow for an exchange of regulators in maturation. Consistent with this, deletion of Blm10 showed no obvious defect in CP maturation (35, 36).

Over the years, many other roles for Blm10 and PA200 have been suggested, including involvement in processes such as spermatogenesis, DNA repair, histone degradation, CP sequestration into proteasome storage granules (PSGs), and degradation of mitochondrial proteins (19, 32, 33, 35, 36, 37, 38, 39, 40, 41). The predicted bromodomain-like region of Blm10/PA200 was shown to specifically bind acetylated histones, which led to the subsequent degradation of histones by PA200-CP complexes (32, 33). However, recent structural work on the PA200-CP complex raises some questions in this regard and shows that PA200 binds to inositol phosphates, which have been shown to bind and regulate histone deacetylases (42, 43). Furthermore, binding of PA200 results in an unusually wide α ring. These conformational changes are propagated to the active site β subunits, thereby changing the structure of the active sites and affecting proteasome activity. Specifically, activation of β2 trypsin-like activity was observed along with a slight inhibition of β5 and β1 activity (44).

In order to gain insight into the physiological role of Blm10 containing complexes, we altered the abundance of this protein in yeast and monitored the effects of its overexpression on the proteasome landscape. Further, we sought to determine how Blm10-bound complexes are affected by starvation conditions.

## Results

### Blm10 overexpression interferes with RP-CP interaction

The proteasome CP is a cylindrically shaped structure able to bind one of numerous regulators. However, binding of these regulators is mutually exclusive as they bind the same interface of CP. Thus, there are a variety of proteasome complexes that contain one or two copies of a particular regulator. Additionally, hybrid complexes (CP with two different regulators) can form. In all, there is the potential for a varied landscape of proteasome complexes. Several of these potential complexes have been observed in yeast as well as mammalian cells, *e.g.* (45, 46); however, our understanding of the mechanisms and regulations that govern this landscape is limited.

**Figure 1:**
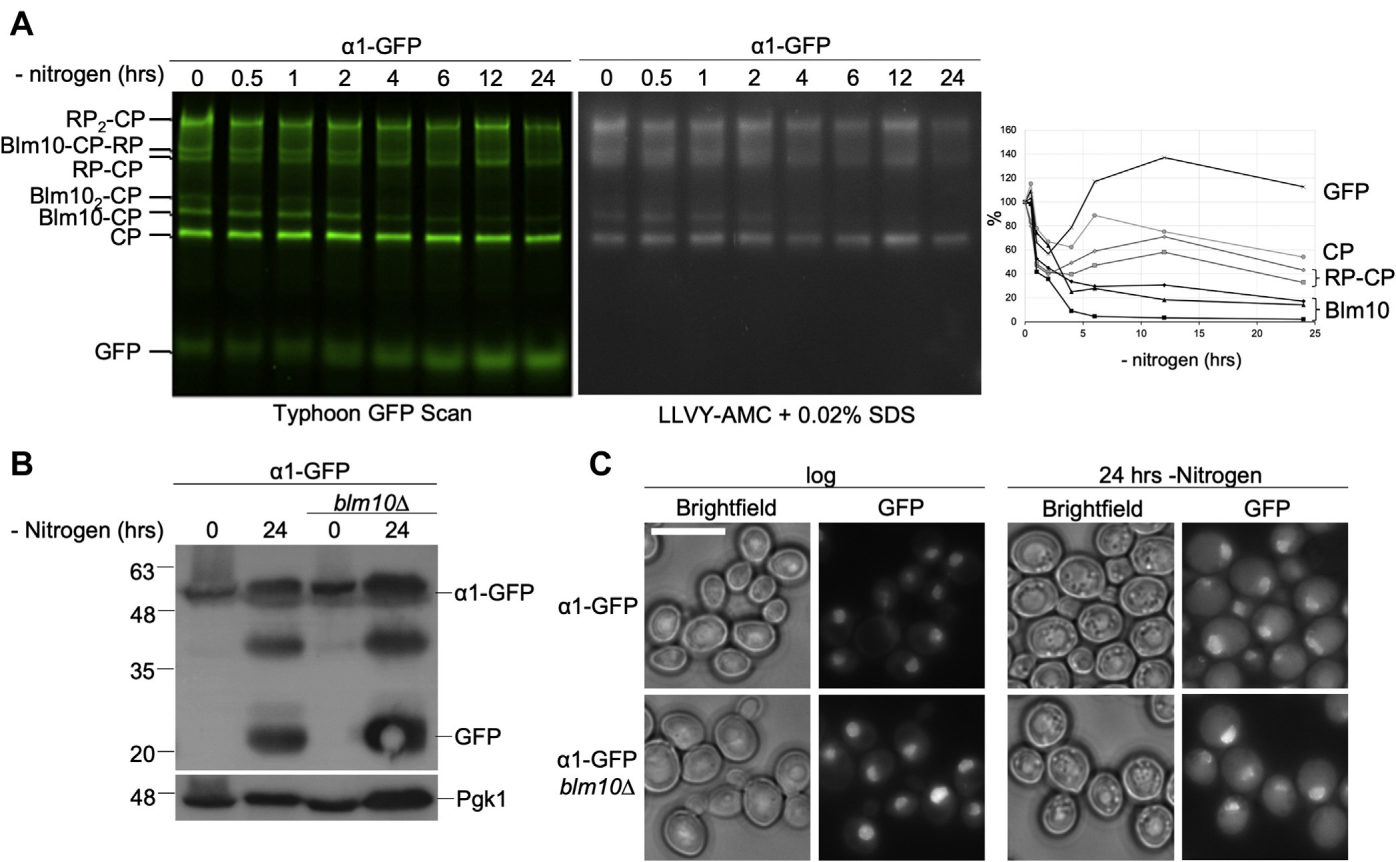
**Blm10-containing complexes are reduced during nitrogen starvation.***A*, strains expressing α1 GFP-tagged proteasomes were analyzed by native gel electrophoresis to determine the effect of nitrogen starvation on proteasome complexes. Total protein lysates were obtained by cryo-grinding and proteasomes were visualized using a Typhoon 9410 scanner. Following the GFP scan, a LLVY-AMC proteasome activity assay was conducted to determine the location of active proteasome complexes (CP bound with an activator). Use of 0.02% SDS opens the CP gate and allows for visualization of proteasome complexes not bound by an activator. Over a 24-h time course, the amount of proteasome complexes was reduced and free GFP was formed. Reduction of Blm10-CP complexes occurred approximately 2 h earlier than what has been established for 26S proteaphagy. Plot shows quantification of GFP scan corrected for loading using an anti-Pgk1 western of SDS-PAGE with equal loading compared with native gel. t = 0 for each proteasome species was set at 100%. “Blm10” indicates the different Blm10-containing complexes (Blm10-CP, Blm10_2_-CP, Blm10-CP-RP). *B*, wild-type and BLM10 deletion strains expressing GFP-tagged α1 from the endogenous locus were lysed before and after nitrogen starvation. Immunoblots for GFP show the formation of free GFP, which indicates vacuolar proteasome degradation through autophagy. Pgk1 (phosphoglycerate kinase) was used as a loading control. *C*, strains as in *B* were analyzed by fluorescence microscopy. GFP localization was monitored in logarithmically growing cells as well as cells grown for 24 h in minimal media lacking nitrogen. Scale bar represents 5 μm.

In lysates of a strain where the endogenous copy of α1 was tagged with GFP, a number of these complexes can be distinguished on native gel (Fig. 1*A*, lane 1). Whole-cell lysate prepared by cryo-grinding was separated on native gel and imaged for GFP to identify the native complexes that contained the CP subunit α1. A number of species can be readily identified; from top to bottom these are: CP with RP bound on both ends (RP_2_-CP), CP-RP with Blm10 bound (Blm10-CP-RP), CP with one RP (RP-CP), CP with Blm10 bound on both ends (Blm10_2_-CP), CP with Blm10 bound on one end, and free CP. Assignment of these species is based on extensive work by us and other laboratories (47, 48, 49). It should be noted that the Blm10-CP-RP band could contain Ecm29, as this proteasome-associated 210 kDa protein can bind to RP-CP, Blm10-CP-RP, and RP_2_-CP complexes. Further, Ecm29 retards the migration of complexes on gel when it is bound (47, 48, 50, 51). Consistent with the fluorescence-based complex assignments, all these bands show hydrolytic activity toward the model peptide substrate LLVY-AMC (Fig. 1*A*, right panel). We have previously shown that nitrogen starvation induces selective degradation of the proteasome through autophagy (proteaphagy). However, cells grown 24 h in yeast peptone dextrose (YPD) or starved for glucose did not lead to selective degradation of RP or CP (52, 53). Here, we noticed the preferred disappearance of Blm10-CP and Blm10-CP-RP species over time (Fig. 1*A*). This disappearance was accompanied by an increase in the amount of a faster migrating GFP band. The migration behavior of this band is consistent with the migration of free GFP on our native gels (53). Free GFP is formed when the GFP is proteolytically cleaved from a tagged protein in the yeast vacuole, but not yet degraded (53, 54). This suggests that either Blm10-containing proteasome species are preferably degraded *via* autophagy or Blm10 dissociates from these complexes upon nitrogen starvation and the free GFP is derived from any form of CP containing complexes that undergo autophagy. Consistent with the latter, *blm10Δ* cells still displayed proteaphagy upon nitrogen starvation (Fig. 1, *B* and *C*, and (55)). Therefore, it is clear that Blm10 is not essential for the autophagy of CP or RP-CP complexes.

**Figure 2:**
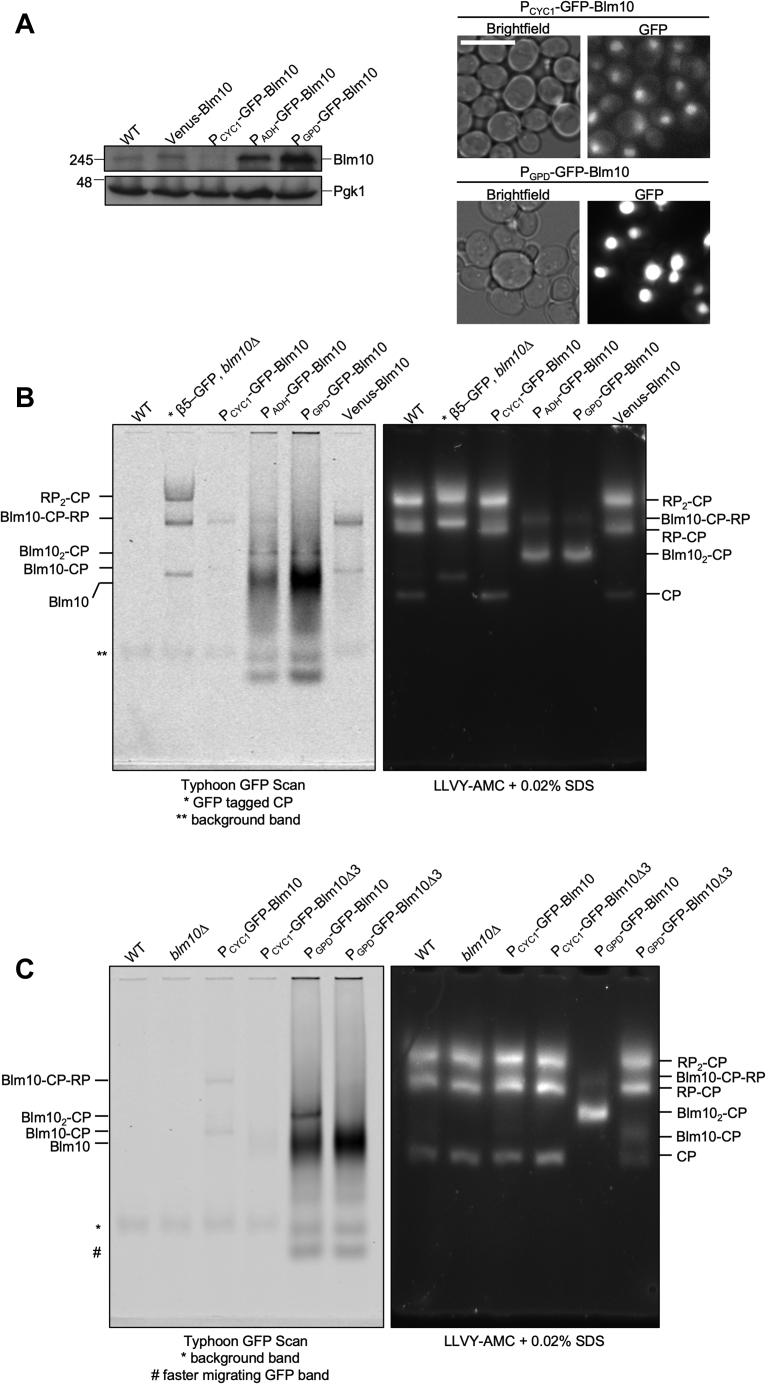
**Overexpression of Blm10 reduces RP-CP levels.***A*, Blm10 was N-terminally tagged with GFP and expression levels were manipulated by changing the endogenous promoter to the *CYC1*, *ADH*, or *GPD* promoters. Total lysate of indicated strains was separated on SDS-PAGE and immunoblotted for Blm10. *Right panel* shows microscopic images for indicated strains. The *CYC1* promoter resulted in expression similar to WT Blm10 levels while the *ADH* and *GPD* promoter resulted in overexpression of the protein. Scale bar represents 5 μm. *B*, native gel electrophoresis of cell lysates from indicated strains was used to determine the effect of Blm10 overexpression on proteasome complexes. Gels were scanned using a Typhoon 9410 imager to visualize GFP-Blm10 containing complexes (*left panel*). Proteasome activity was analyzed using the fluorogenic peptide substrate LLVY-AMC (*right panel*). *C*, cell lysates from indicated strains were compared with mutant versions of Blm10 lacking the C-terminal tail (YYA). The tail mutation prevented binding of Blm10 to CP even upon strong overexpression as shown by native gel electrophoresis.

As such, it is important to understand how the landscape of CP containing proteasome complexes is controlled by the protein levels of Blm10 in yeast and whether Blm10-containing complexes behave differently than RP-containing complexes under stress conditions such as nitrogen and glucose starvation. Although Blm10 overexpression has been studied previously (30, 49), there have been some conflicting reports regarding its impact on growth. Furthermore, some studies were conducted with, or included strains, where Blm10 was tagged C terminally (19, 35, 55). However, C-terminal tagging renders Blm10 nonfunctional as the C terminus of Blm10 is essential for its interaction with CP (11, 30). Here, we introduced an N-terminal fluorescent tag on *BLM10* at its endogenous locus with different promoters. We either introduced the *CYC1*, *ADH*, or *GPD* promoters (56), which provide increasing levels of Blm10 expression or retained the endogenous promoter using a Cre-Lox-based approach. The expression level we observed with the CYC1 promoter was similar to the levels of Blm10 we observed with the endogenous promoter under conditions of logarithmic growth (Fig. 2*A*, lanes 1–3). Replacement with either the ADH or GPD promoter resulted in strongly increased levels of Blm10 in total lysate immunoblots and an eightfold (ADH) to 12-fold (GPD) higher fluorescence intensity as compared with the endogenously expressed GFP-Blm10 (Fig. 2*A*, lanes 4 and 5). Regardless of the expression level, the signal for GFP-Blm10 remained predominantly nuclear (Fig. 2*A*).

Compared with CP, endogenous Blm10 levels are substoichiometric, and any increase in Blm10 could lead to an accumulation of free Blm10, more Blm10 associated with CP, or both. That said, we are not aware of any publications showing the presence of wild-type Blm10 that is not associated with proteasomes in cells. Therefore, to test how increased Blm10 expression influenced the proteasome landscape, we analyzed lysates expressing different levels of Blm10 on native gel. A β5-GFP expressing strain was used as a control to compare CP containing complexes. The β5-GFP tag resulted in a slight shift of CP species on the native gel (Fig. 2*B*, lane 2). As expected, in cells that express GFP-Blm10 at levels similar to wild-type (CYC1 promoter), the landscape consisted mostly of RP_2_-CP, RP-CP, and CP complexes. Some RP-CP-Blm10 complexes were also visualized on native gel as indicated by LLVY-AMC active species (Fig. 2*B*, right panel lane 3), which is very similar to wild-type cells (lane 3 compared with 1 and 6). The fluorescent scan of the same gel shows the major band of RP-CP-Blm10 and a faint Blm10-CP species. With increased expression of Blm10 (lanes 4 and 5), we observed a dramatic loss of the RP-CP and RP_2_-CP species with only small amounts of RP-CP-Blm10 remaining. Most CP, however, could be found in a species hardly detectable in wild-type cells. Scanning the gel for GFP fluorescence (Fig. 2*B*, left panel), we observed GFP-Blm10 migrates at the same location as this species. Based on the shift, this band represents CP with both faces of the complex occupied with Blm10 (Blm10_2_-CP) (49). Interestingly, Blm10 levels appeared to be in excess of CP-binding surfaces and accumulated free GFP-Blm10 could be observed on the native gel. Consistent with promoter activity, more free GFP-Blm10 was observed with the GPD promoter than ADH promoter. In sum, Blm10 is a substoichiometric CP regulator that is normally found associated with a subset of CP. Increased expression resulted in reduced levels of free CP and 26S and led to an accumulation of free Blm10, which was in excess of the available CP-binding surfaces.

As free Blm10 has never been reported on native gels, we wanted to confirm our assignment of free Blm10 by comparing Blm10 mutant strains that are defective in CP binding. Based on the crystal structure of Blm10-CP, the C-terminal tail is important for its interaction with CP and deletion of the last three amino acids of Blm10 has been shown to disrupt interactions between CP and Blm10 (11, 30). With the *Blm10Δ3* mutant strain, we observed that even the strong overexpression of this mutant was not able to reduce the amount of RP-CP species normally found in yeast (Fig. 2*C* right panel, lane 5 compared with 6). Scanning the gels for bands that show GFP fluorescence, we observed that the Blm10_2_-CP band was absent in the lysate from *Blm10Δ3* cells while the other major band remained indicating this was indeed free GFP-Blm10 that migrated here on native gel (Fig. 2*C*, left panel). As there were no detectable levels of free Blm10 under endogenous expression (Fig. 2*B*, lanes 3 and 6), increasing the levels of Blm10 appeared to directly affect the number of Blm10-CP complexes present in the cell as well as 26S proteasomes.

Surprisingly, overexpression of Blm10 also resulted in the appearance of a faster migrating band (Fig. 2*C*, labeled #), which was unaffected by the deletion of the last three amino acids of Blm10. This band migrates at a position consistent with free GFP (see *e.g.*, Fig. 1*A* or (53)). However, it should be noted that the migration of complexes on these native gels not only depends on the molecular weight of the complex, but also on the size and shape. As such, there is not always a correlation between migration behavior of a complex and the molecular weight. Considering that Blm10 is known to bind to immature forms of CP (35, 57), this band could also represent Blm10 bound to immature CP. For example, overexpression of *PBA1-PBA2* causes an accumulation of the immature 15S complex that migrates faster than CP on native gel (34) and Pba1-Pba2 binding to immature CP is structurally different as compared with mature CP. If this is similar for Blm10, the interaction with immature CP might depend less on the Blm10 Hb-Y-X motif (18).

RP is normally not found associated with immature CP because Pba1-Pba2 prevents this interaction. One possibility for the apparent increase in Blm10-CP complexes is that Blm10 binds to immature CP and has an extremely low off-rate resulting in an inability of RP to compete with Blm10 for CP binding. To test if the overexpression of Blm10 caused excessive binding of Blm10 to immature CP, we sought to determine if Blm10 could displace the chaperone dimer Pba1-Pba2, which binds to the same interface of immature CP as RP does to CP (13). To assess changes in the levels of Pba1-2 bound to immature CP under conditions of Blm10 overexpression, we purified mature and immature CP from strains with endogenous or strongly overexpressed *BLM10*. Purified mature CP from cells that overexpressed Blm10 showed reduced levels of the RP subunit Rpn8 and increased levels of Blm10, indicating that Blm10 competed with RP for CP binding (Fig. S1*A*, lane 4). These results are consistent with our observations of whole-cell extracts separated by native gel. Purified immature CPs, however, showed only slightly less Pba1-2 in the presence of increased levels of Blm10 (Fig. S1*A*, lane 2). To test if the Blm10 tail was expendable for Blm10-immature CP binding, as would be expected by a lack of reduction for the faster migrating species in Figure 2*C*, we next evaluated the impact of Blm10Δ3 expression on the purification of immature CPs. Purified immature CP showed reduced levels of Blm10 binding, both under the endogenous and GPD promoter, in strains expressing Blm10Δ3. This shows that the Hb-Y-X motif of Blm10 is critical for both CP and immature CP binding, suggesting that Blm10 interactions with the alpha ring of immature CP are similar to that of mature CP (Fig. S1, *B* and *C*). This appears different than Pba1-2, which becomes embedded into immature CP but not mature CP (13, 34). Furthermore, the faster migrating species we observed at the bottom of the native gel in Figure 2*C* is not Blm10 bound to immature CP, but likely represents a small amount of free GFP derived from low levels of autophagy. Alternatively, it could pertain to Blm10-immature CP species that do not contain Ump1, as we used Ump1-TAP tag to purify immature CP and thus limited the immature CP forms we could analyze to Ump1 containing complexes.

### Phenotypic analysis of Blm10 overexpression

**Figure 3:**
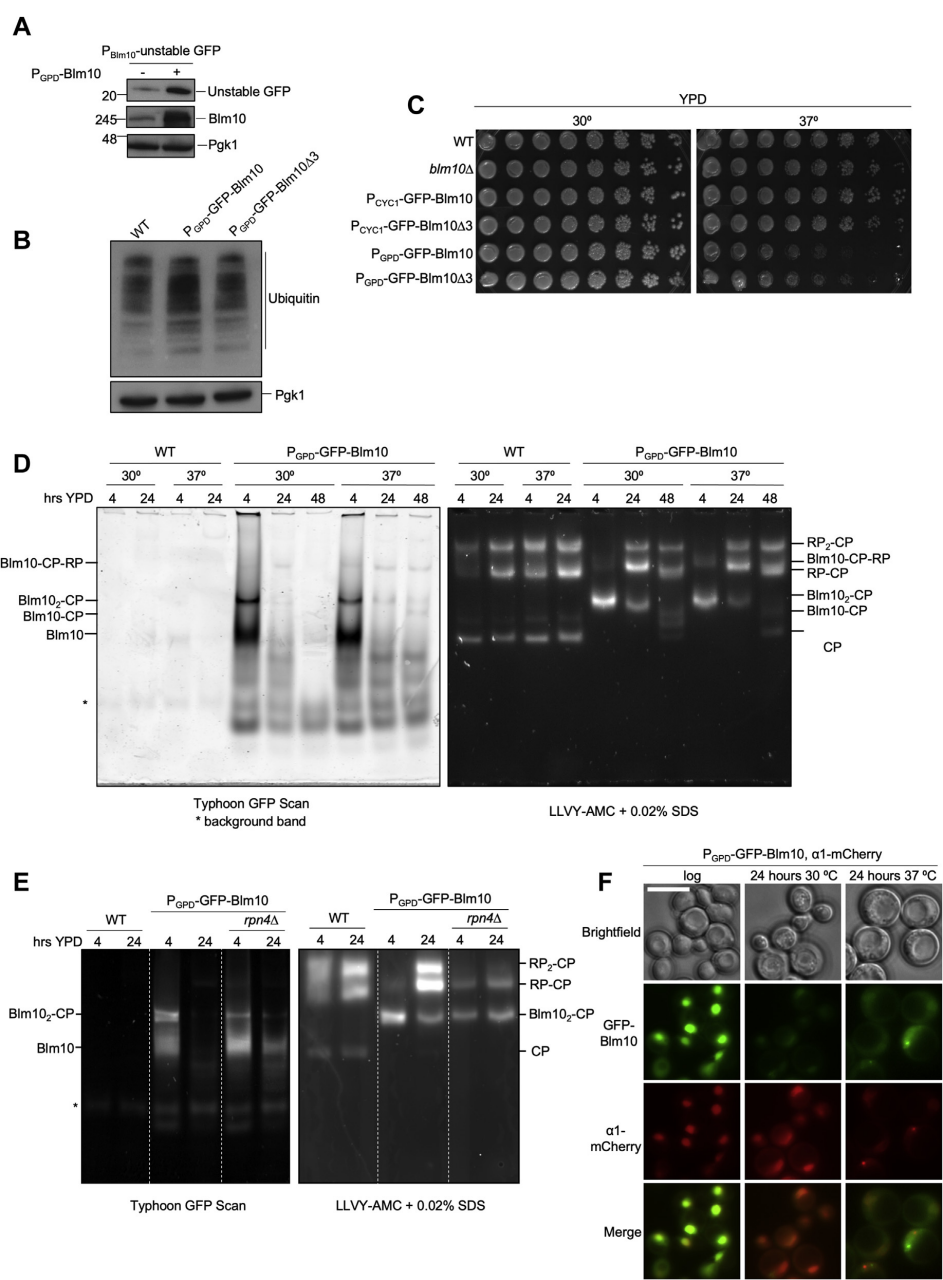
**Overexpression of Blm10 leads to proteasomal substrate accumulation.***A*, unstable GFP was expressed in wild-type cells or Blm10 overexpressing cells from the GPD promoter. Cell lysates were separated on SDS-PAGE and immunoblotted for indicated proteins. Elevated levels of unstable GFP upon Blm10 overexpression were observed in three independent experiments. Pgk1 was used as a loading control. *B*, wild-type cells, Blm10, or Blm10Δ3 overexpressing cells were lysed, and lysates were separated on SDS-PAGE and immunoblotted for indicated proteins. An increase in overall ubiquitinated material resulted upon overexpression of WT Blm10 while no such increase was observed upon overexpression of the noncompetitive mutant of Blm10, which cannot bind CP. *C*, indicated strains were serially diluted following logarithmic growth and spotted on YPD plates to determine if Blm10 overexpression resulted in decreased cell survival. Plates were incubated at 30 °C and 37 °C for 48 h. At 30 °C, all strains grew similarly, but at elevated temperatures, a modest growth phenotype was associated with overexpression of Blm10, which was partly rescued by the mutant version of Blm10. *D*, whole-cell lysates from cells grown at 30 °C and 37 °C were resolved using native gel electrophoresis. GFP-Blm10 species were visualized using a Typhoon scanner while an in-gel LLVY-AMC assay was conducted to determine the location of active proteasome complexes. Blm10 levels were reduced over time and 26S proteasomes had re-formed, which likely explains the modest growth phenotype observed in *C*. *E*, indicated yeast strains were grown in YPD to log phase, then harvested by centrifugation. Cells were lysed and proteins analyzed by native gel as described above. *F*, fluorescent microscopic analyses of cells from overexpressing GFP-Blm10 and α1-mCherry. Consistent with the native gel, GFP-Blm10 levels were reduced over time as determined by fluorescence microcopy; α1-mCherry localization at 30 °C remained nuclear, while at 37 °C, α1-mCherry formed granules that were devoid of GFP-Blm10. Scale bar represents 5 μm.

With the exception of Rpn10, Rpn13, Sem1, Rpn9, and α3, all CP and RP subunits are essential, which is consistent with the important role for RP-CP complexes in the degradation of ubiquitinated proteins. Therefore, we anticipated that the very low level of RP-CP-Blm10 proteasomes we detected, together with the absence of 26S proteasomes in Blm10 overexpressing strains (Fig. 2*B*), would not suffice to maintain normal cellular proteostatic functions. To test the effect of Blm10 overexpression on proteasome substrates, we expressed an unstable N-end rule GFP substrate in a strain overexpressing Blm10. In cells containing 26S proteasomes, unstable GFP is present at a low steady-state level (Fig. 3*A*, lane 1). Overexpression of Blm10 increased these levels by almost 3-fold (2.8 ± 0.58 SEM) indicating a strongly reduced degradative capacity in these cells (Fig. 3*A*, lane 2). To determine if the reduced degradation expanded beyond this model substrate, we examined overall levels of ubiquitinated material by blotting for ubiquitin in cell lysate. Logarithmically growing cells containing high levels of Blm10 showed a trend of increased accumulation of ubiquitinated material; however, this was modest and upon quantification of four independent repeats not a statistically significant difference (Fig. 3*B*, compare lanes 1 and 2). Thus, the degradation of an overexpressed unstable model substrate is clearly impacted, but the low levels of 26S proteasomes resulting from Blm10 overexpression (as seen on native gels for RP-CP and RP_2_-CP) appear to cause only a modest reduction in the ability of cells to degrade ubiquitinated substrates. Consistent with this, the strains overexpressing Blm10 were viable and did not show any apparent growth defects on YPD plates or in liquid culture for logarithmically growing cells (Fig. 3*C*). In fact, cells grew at normal rates and even better than an *rpn4*Δ strain. *rpn4*Δ cells cannot upregulate proteasome levels and have lower proteasome levels than wild-type cells. Nevertheless, the *rpn4*Δ strain retained higher basal proteasome levels as compared with Blm10 overexpression (58). These data suggest that, under these nonstress conditions, low proteasome levels are sufficient for survival. This also indicates that the slow growth of *rpn4*Δ in rich media under logarithmic growth conditions is not exclusively due to lower expression of proteasomes. Indeed, Rpn4 regulates the expression of numerous other proteins (59, 60, 61), some of which contribute substantially to the slow growth phenotype. Alternatively, Blm10_2_-CP could compensate for, or replace, the 26S degradative capacity. However, the latter is unlikely considering that Blm10, unlike many proteasome subunits, is not essential as it lacks ubiquitin receptors and does not have the ability to utilize ATP to unfold and translocate proteins into the degradative chamber of CP.

Compromised proteasome function or reduced proteasome levels, as has been observed with many mutants, is associated with increased sensitivity of strains to high temperature or in the presence of the arginine analog canavanine. Under these conditions, protein misfolding and unfolding increase the demand for proteasomal degradation. Considering the low levels of 26S in the strain overexpressing Blm10, we expected to see a strongly reduced growth or survival for this strain compared with wild-type under these conditions. However, the GPD promoter-driven overexpression of Blm10 resulted in only a modestly reduced growth phenotype at 37 °C and no detectable difference in the presence of canavanine (Figs. 3*C* and S2*A*). However, overexpression of Blm10 resulted in reduced petite formation (small colonies due to loss of mitochondrial DNA) compared with wild-type following acute heat stress at 42 °C (Fig. S2*B*). This is interesting considering that the deletion of *BLM10* causes increased petite formation (11) and indicates physiological relevance as overexpression causes the opposite effect. Moreover, the overexpression of *Blm10Δ3*, which is compromised in CP binding and has normal 26S proteasome levels, resulted in an almost similar reduction in growth as the Blm10 overexpression, indicating it is more likely the high levels of Blm10 itself (*i.e.*, independent of its ability to reduce 26S proteasome levels) that are responsible for the phenotype (Fig. 3*C*).

To understand why overexpression of Blm10 produced only a modest growth phenotype, we looked at the proteasome landscape for wild-type and Blm10 overexpressing strains under the conditions tested. For wild-type cells, the landscape of proteasome complexes remained similar when comparing growth in YPD over time (4–24 h) or at 30 °C and 37 °C (Fig. 3*D*). As mentioned earlier, upon Blm10 overexpression, the landscape was comprised of mainly Blm10_2_-CP complexes and a small amount of Blm10-CP-RP at 30 °C. A similar composition was also observed in logarithmically growing cells at 37 °C. However, growth of cells under these conditions for 24 or 48 h induced dramatic changes in the proteasome composition as observed on native gel. At 24 h, the levels of Blm10_2_-CP were reduced, Blm10-CP-RP increased, and RP_2_-CP was detectable at both temperatures (Fig. 3*D*, lanes 6 and 9 on right).

To determine if the re-emergence of RP-CP complexes was due (in part) to interactions with existing free RP in the cells, we analyzed a strain where Rpn1 was tagged with mCherry at the endogenous locus and GFP-Blm10 was overexpressed. We observed free RP in logarithmically growing cells, indicating that free RP complexes are present, but the excess of Blm10 prevented all RP from associating with CP. At 24 h, as Blm10 complexes decreased, RP-CP levels increased, and the levels of free RP were undetectable (Fig. S3*A*). This suggests that free RP complexes are now able to associate with CP. However, the levels of free RP do not seem sufficient to explain the increase in RP-CP complexes. Indeed, when we used an *RPN4* knockout strain to evaluate the need for this proteasome transcription factor in the process, we observed that the production of new proteasome components is important to re-establish RP-CP complexes. In the *rpn4Δ* strain, the levels of Blm10-CP and RP-CP complexes were similar at both 4 h and 24 h (Fig. 3*E*). Intriguingly, free Blm10 levels were only slightly reduced (Fig. 3*E*, left panel, compare lanes 5 and 6). One caveat here is that an Rpn4 deletion results in reduced proteasome levels, so the levels of free RP that could reassociate with CP will also be reduced. Consistent with this, *rpn4Δ* with Blm10 overexpression resulted in increased temperature sensitivity (Fig. S3*B*). In all, it is likely that both an increase in the levels of RP and the utilization of a pool of preexisting, free RP is responsible for the decline in Blm10 association with CP as Blm10 levels are reduced.

Consistent with the native gel analyses, microscopic analyses of cells at 37 °C showed reduced levels of GFP-Blm10 compared with 24 h at 30 °C where we detect GFP-Blm10 in the nucleus. Intriguingly, the 37 °C stress response led to formation of proteasome granules that lacked GFP-Blm10 (α1-mCherry in Fig. 3*F*). This was surprising as previous reports have shown that Blm10 and proteasomes colocalize in granules in stationary phase and upon carbon starvation (55, 62). The formation of these granule structures is thought to protect proteasomes from degradation, so failure of Blm10 to localize to these granules could lead to its reduced levels. However, apart from degradation, a reduction in Blm10 levels could also be caused by reduced transcription or translation and protein dilution resulting from cell division. To test how stable and unstable proteins behave when driven by this promoter under these conditions, we introduced the same GPD promoter upstream of an open reading frame encoding a stable or an unstable form of GFP. The newly generated strains were subjected to the same conditions as the Blm10 overexpressing strains. Here, the cells expressing stable GFP maintained constant levels of GFP at both 30 °C and 37 °C for up to 24 h of growth (Fig. S4*A*), while for the unstable GFP, the equilibrium between expression and degradation resulted in a reduction of GFP levels, similar to what we saw for Blm10. Similar results were obtained under nitrogen and glucose starvation (Fig. S4*B*). Interestingly, there is a slightly faster migrating band observed for stable GFP. This band was not derived from proteasomal degradation, as it was still observed for cells treated with MG132 (Fig. S4*B*, right panel, lanes 8 and 10). Instead, bulk autophagy induced with these conditions targets some of the stable GFP to the vacuole, where the GFP core is resistant to degradation, but C and N-terminal extensions can be trimmed (63). In all, these data suggest that the promoter activity is reduced under the stress conditions with nitrogen starvation causing a stronger reduction in expression than other conditions. Nevertheless, the data for stable GFP show that this reduced activity cannot explain the observed reduction in protein level through simple dilution by cell division, as stable GFP levels remain constant. We also compared stable and unstable GFP driven from the *BLM10* promoter under both nitrogen and glucose starvation (Fig. S4*C*). This promoter clearly has lower activity, but levels of stable GFP were maintained under nitrogen and glucose starvation. In all, these data indicate that the reduction of Blm10 under the conditions tested cannot be explained simply by a lack of expression from the GPD promoter and indicates that the strong reduction in Blm10 levels occurs due to active degradation.

### CP-bound Blm10 is degraded by autophagy

**Figure 4:**
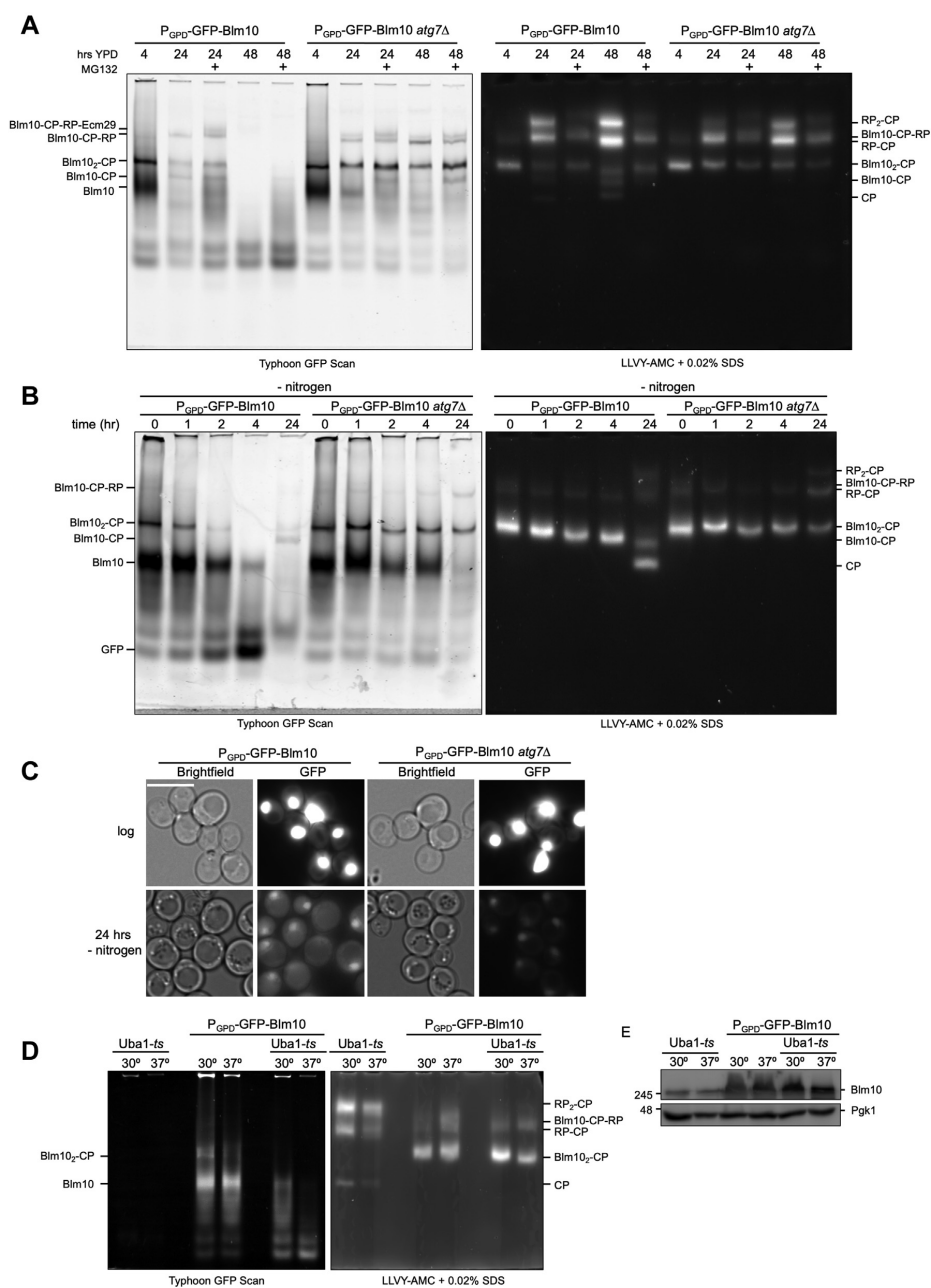
**Blm10 is selectively degraded through autophagy.***A*, GFP-Blm10 levels were monitored over a 48-h time course in WT and *atg7Δ* strains in the presence of the proteasome inhibitor MG132 (100 μM). Cell lysates were separated using native gel electrophoresis. Blm10-bound CP complexes showed stabilization in strains lacking Atg7 while proteasome inhibitor had little impact on Blm10 levels. *B*, samples were analyzed as in (*A*) only following nitrogen starvation. *ATG7* deletion resulted in stabilized Blm10-bound complexes in nitrogen-starved cells similar to cells grown in YPD for 24 and 48 h. *C*, strains overexpressing Blm10 were analyzed using fluorescent microscopy. *atg7Δ* cells were consistently devoid of GFP signal in the vacuole while WT strains showed GFP inside the vacuole. This indicates that GFP-Blm10 complexes are targeted for autophagy upon nitrogen starvation. Scale bar represents 5 μm. *D*, indicated yeast strains were inoculated in YPD at OD 0.5. Cells were grown at 30 °C for 4 h or grown for 2 h at 30 °C, followed by 2 h at 37 °C. Equivalent of 50 ODs of cells were cryo lysed and analyzed by native gel electrophoresis. *E*, lysates from (*D*) were separated on an 8% SDS-PAGE, then transferred to a PVDF membrane, and blotted for Blm10 and Pgk1.

As our data suggest that reduction of Blm10 results from degradation, we next wanted to determine the degradative pathway responsible. Since the majority of protein degradation depends on either the autophagy-lysosome or the ubiquitin-proteasome pathway, we tested for the involvement of either system in the reduction of Blm10. We first used the proteasome inhibitor MG132 (100 μM) to test the extent to which Blm10 reduction was dependent on proteasome activity. In cells overexpressing Blm10, the addition of proteasome inhibitor did not prevent the strong reduction of Blm10_2_-CP complexes observed after growth for 24 or 48 h in YPD (Fig. 4*A*). The band observed directly above Blm10-CP-RP in the proteasome inhibitor-treated cells resulted from the association of Ecm29 with these complexes. Since *ECM29* has the PACE element in its promoter, it is recognized by Rpn4 and upregulated in several strains with compromised proteasome function (47, 64, 65, 66, 67). Therefore, upregulation of Ecm29 upon proteasome inhibition is expected. Ecm29 associates with RP-CP containing complexes as it has a binding site on RP as well as CP (47, 66, 67). In all, proteasome inhibitor treatment did not block the reduction of Blm10-CP complexes.

Previous work on yeast has shown that proteasomes (RP and CP) are degraded through autophagy under nitrogen starvation or upon proteasome inhibition (52, 53, 68). To test for an autophagic contribution in reshaping the proteasome landscape, we deleted *ATG7*. *ATG7* encodes for a protein that is required in both micro- and macroautophagy as it activates the ubiquitin-like proteins Atg8 and Atg12, both of which are crucial for autophagosome formation (69). In the *ATG7* knockout strain, we also observed a reduction in the levels of free Blm10; however, the levels of Blm10 that were complexed with the CP were largely stabilized with hardly any reduction in Blm10-CP complexes even after 24 h. This was in contrast to the autophagy-capable cells, where barely any Blm10-CP complex was detected (Fig. 4*A*). This indicates that Blm10-CP complexes are degraded through autophagy upon extended growth in YPD. The autophagic degradation is interesting under these conditions as it corroborates our original observations under nitrogen starvation (Fig. 1*A*) as well as previous work showing that proteasomes (RP and CP) are degraded through autophagy under nitrogen starvation or upon proteasome inhibition (52, 53, 68).

Next, we tested if Blm10 levels would be affected similarly by nitrogen starvation as compared with prolonged growth. The reduction of Blm10 in wild-type cells began approximately 2 h after starvation and steadily decreased with little to no detectable free Blm10 after 24 h (Fig. 4*B* lanes 1–5). In the *ATG7* knockout strain, free Blm10 levels remained steady up to 4 h of nitrogen starvation and were not completely cleared after 24 h. This indicates that free Blm10 was partially stabilized in the autophagy defective strain. Similar to prolonged growth, the CP-bound Blm10 levels also remained almost constant showing only a slight reduction at 24 h (Fig. 4*B*, lane 10). Consistent with the autophagic degradation of Blm10, we observed the accumulation of free GFP on native gels after nitrogen starvation in an autophagy-dependent fashion (Fig. 4*B*, see 4 h wt *versus atg7*Δ). Furthermore, fluorescence could be observed in the vacuole of WT cells overexpressing Blm10, but not when *ATG7* was deleted (Fig. 4*C*). Here, GFP-Blm10 was found in the nucleus and the vacuoles were void of fluorescent signal as reported previously (35, 49). Thus, Blm10 vacuolar targeting and degradation are mediated by autophagy, and both prolonged growth and nitrogen starvation induce the autophagic degradation of Blm10-bound CP complexes. GFP-labeled Blm10 showed a smearing pattern on native gel (Fig. 4*B*, lanes 1–3). As Blm10 is degraded and both proteasomal and autophagic degradation often involves substrate ubiquitination, we wondered if this reflected Blm10 ubiquitination. To test this, we used a *uba1*-*ts* strain for which ubiquitination is compromised at the nonpermissive temperature (70, 71). In *uba1*-*ts* cells overexpressing Blm10, we observed a smear at 30 °C during logarithmic growth. However, when switched to the nonpermissive 37 °C for 2 h, there was a strong reduction in the Blm10 smear we observed on the gel (Fig. 4*D*). Similarly, when we immunoblotted lysates separated on SDS-PAGE, we observed reduced signal at a molecular weight slightly above 246 kDa with the *uba1-ts* strain at the nonpermissive temperature. Altogether, this suggests that free Blm10, *i.e.*, not CP-bound, was ubiquitinated, presumably as part of the degradative process.

### Free Blm10 is degraded by both autophagy and the proteasome

**Figure 5:**
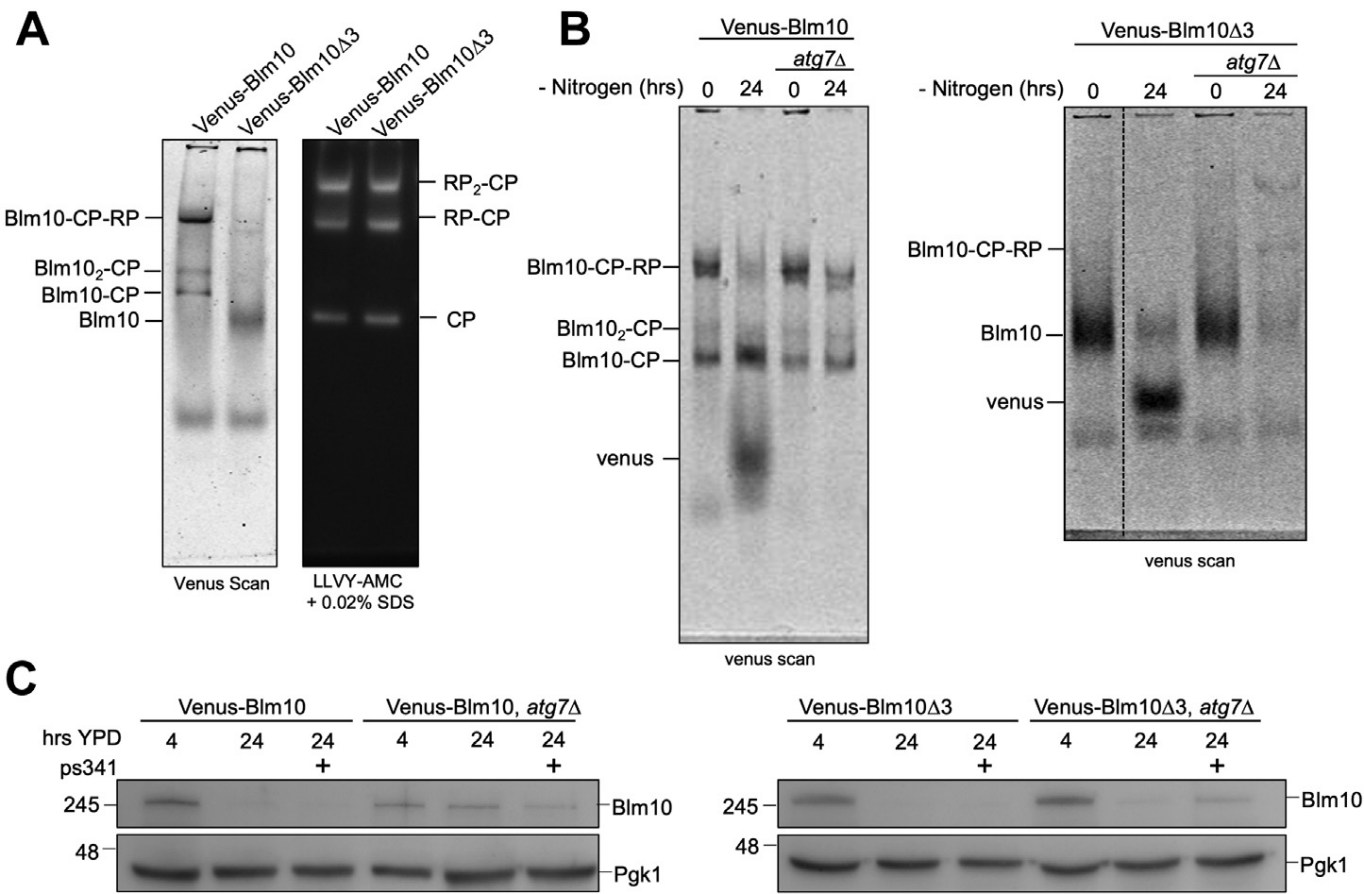
**Blm10 is degraded by the proteasome when autophagy is blocked.***A*, strains expressing Venus-Blm10 or Venus-*Blm10Δ3* under the endogenous promoter were lysed under native conditions and lysate was separated on native PAGE. Gels were analyzed by Typhoon 9410 scanning for Venus (*left panel*) and LLVY-AMC proteolytic activity (*right panel*). Deletion of the C-terminal tail of Blm10 (amino acids YYA) prevents its ability to bind CP. *B*, native gel electrophoresis of lysates of indicated strains before and after nitrogen starvation. *Dotted line* in *right panel* indicates a break in the same gel (one lane in between was cropped out). The appearance of free Venus indicates vacuolar degradation of Venus-Blm10. Blocking autophagy (*atg7Δ*) prevented the formation of free Venus and both Blm10-bound complexes and free Blm10 were stabilized. *C*, SDS-PAGE separation and immunoblot analysis of Blm10 levels following 24 h growth in YPD indicated bound Blm10 was stabilized when autophagy was blocked, but free Blm10 was not. Free Blm10 was degraded through autophagy, but when this pathway was blocked, it was instead degraded by the proteasome as levels were modestly stabilized in the presence of proteasome inhibitor, PS341 (100 μM). Pgk1 was used as a loading control.

While the data above show that Blm10 is targeted for vacuolar degradation by autophagy, this was under conditions of artificial overexpression of Blm10. To test Blm10 degradation when the protein was expressed from the endogenous promoter, we utilized the Venus-Blm10-tagged strain described earlier. To determine if changes in protein level were dependent on CP binding (all endogenously expressed Blm10 is CP-bound), we also deleted the C-terminal three amino acids (YYA) resulting in free Blm10 (*i.e.*, not associated with CP, Fig. 5*A*). First, we determined whether the levels of bound or free Blm10 changed upon nitrogen starvation or proteasome inhibitor treatment. Upon nitrogen starvation, both strains showed the appearance of free Venus together with a reduction in CP-bound Blm10 (left panel) and free Blm10 (right panel) (Fig. 5*B*). In strains defective in autophagy (by the deletion of *ATG7*), the formation of free Venus was prevented indicating that Blm10 was at least, in part, degraded *via* autophagy. In the presence of Atg7, Venus was cleaved from free Blm10; however, in the *atg7Δ* strain we still saw a reduction in free Blm10, with no accumulation of Venus (Fig. 5*B*, right panel, lane 2 *versus* 4). This suggests that clearance of unbound Blm10 is not mediated solely by autophagy but can presumably be degraded by the proteasome as well. Further, we observed higher migrating bands for Blm10Δ3 in the *atg7*Δ strain following nitrogen starvation. While we are not certain of the nature of these bands, one band is consistent with the migrating pattern for GFP-Blm10-CP-RP, suggesting that the Venus-Blm10Δ3 under these conditions might be able to associate with CP. This might be possible as not all affinity between Blm10 and CP is lost upon the deletion of the last three amino acids from Blm10. As shown earlier we purified a small fraction of Blm10Δ3 with immature CP (Fig. S1, *B* and *C*). Furthermore, nitrogen starvation disrupts interactions between RP and CP, which might allow for more Blm10Δ3 to associate despite its reduced affinity for CP (53, 68).

To test if proteasome activity was responsible for the decrease in Blm10 levels for cells grown for 24 h in YPD, we next grew endogenously expressing Blm10 cells in the presence or absence of proteasome inhibitor. As expected, based on our data with GFP-Blm10 overexpression, Venus-Blm10 levels were reduced. This reduction of Blm10 was not prevented by proteasome inhibitor treatment but was limited in a strain defective for autophagy (Fig. 5*C*, left panel). For unbound Blm10, we observed a similar reduction in levels for strains where autophagy was functional and these levels were also not stabilized with the addition of proteasome inhibitor indicating degradation is primarily through the autophagy pathway (Fig. 5*C*, right panel, lane 3). However, in the *ATG7* knockout, we still saw a reduction of Blm10Δ3, which, to some extent, was stabilized with the addition of proteasome inhibitor (Fig. 5*C*, right panel, lane 6). Thus, unbound Blm10 was degraded by both autophagy and the proteasome, while proteasome-bound Blm10 was largely cleared from cells *via* autophagy.

### Overexpression of Blm10 does not alter proteasome storage granule dynamics

In addition to autophagy, proteasomes and proteasome-associated proteins are also regulated through relocalization. Under certain conditions, proteasomes are sequestered into cytoplasmic granules termed proteasome storage granules (PSGs) (72). Previous reports indicate that Blm10 targets CP to PSGs (19, 55) which have been proposed to provide a protective mechanism against autophagy (55); however, we failed to observe Blm10 in CP containing granules at 37 °C (Fig. 3*F*) suggesting that granules formed under this stress might involve different targeting mechanisms. Therefore, we evaluated to what extent Blm10 is involved in other granule forming conditions such as glucose starvation as well as how Blm10 itself is affected by these stress conditions. As shown previously, Blm10 is found in the nucleus of logarithmically growing cells. Our data show that Blm10 is able to enter the nucleus independent of CP as conditions where most Blm10 is not bound to CP (strong overexpression or Blm10Δ3 truncation) showed Blm10 predominantly in the nucleus (Fig. S5*A* and (55)). Indeed, many Blm10 orthologs contain a canonical nuclear localization signal (NLS) within the C-terminal region of the protein. While bioinformatic analyses of Blm10 (*S. cerevisiae*) did not show a canonical NLS, deletion of the C-terminal region resulted in cytosolic localization of Blm10 (41, 49). This suggests that *S. cerevisiae* Blm10 has a noncanonical NLS.

**Figure 6:**
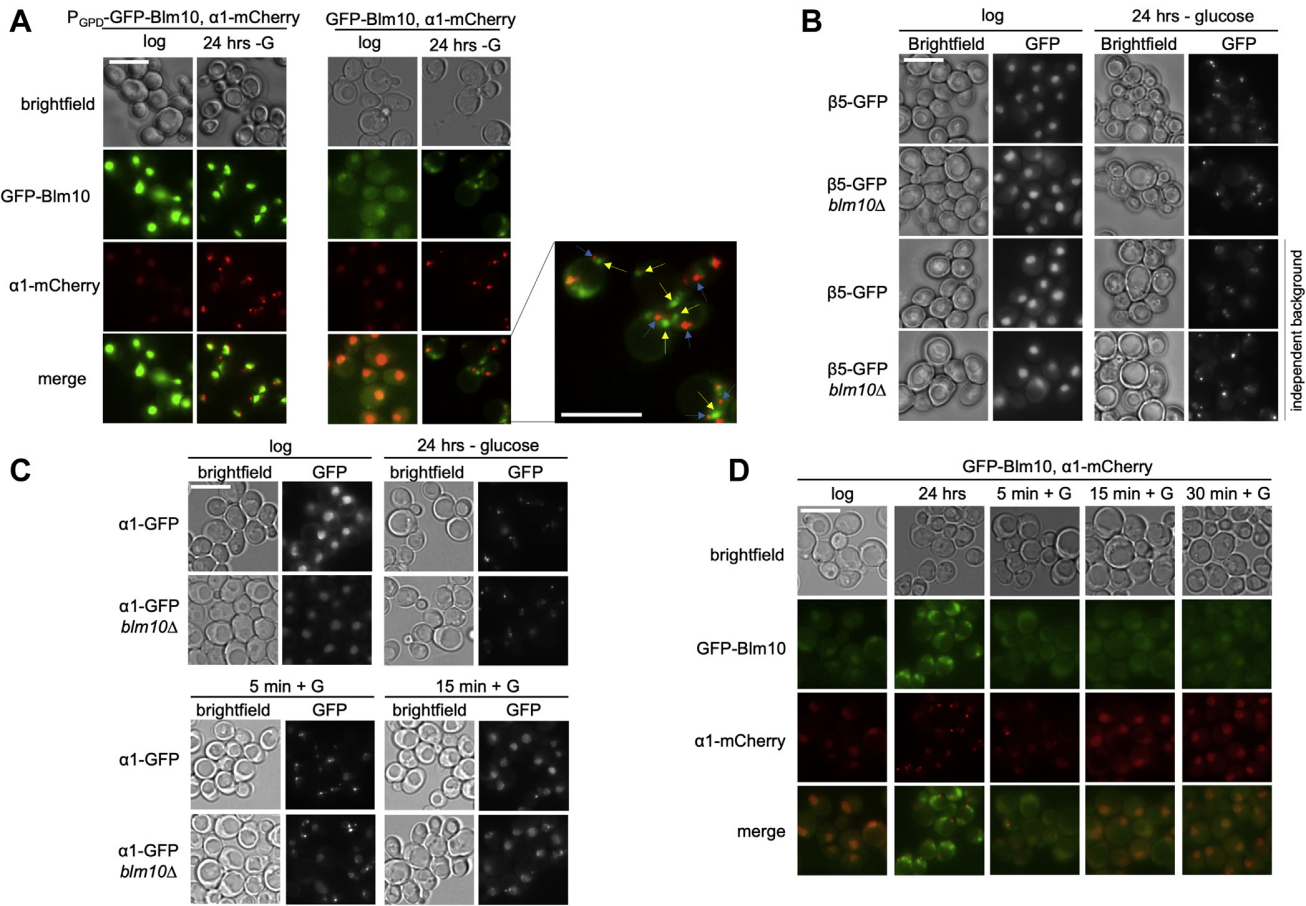
**Blm10-independent formation of proteasome containing granules following glucose starvation.***A*, *left panel*: strains overexpressing GFP-Blm10 and α1-mCherry were visualized using fluorescence microscopy in logarithmically growing cells and following 24 h of growth in media lacking glucose. GFP-Blm10 remained nuclear following glucose starvation where α1-mCherry formed proteasome storage granules (PSGs). The lack of GFP-Blm10 in these granules indicates that α1-mCherry forms glucose granules independently of GFP-Blm10. *Right panel*: similar results were observed with GFP-Blm10 under its endogenous promoter. α1-mCherry again formed PSGs independent of Blm10 while Blm10 signal was seen both disperse in the cytoplasm and forming granule-like structures. Enlarged, merged image shows independent Blm10 granule-like structures (*yellow arrows*) and α1-mCherry PSGs (*blue arrows*). Scale bar represents 5 μm. *B*, two sets of strains (this study and (19, 62)) expressing β5-GFP but lacking Blm10 were monitored for granule formation using fluorescence microscopy following 24 h of glucose starvation. Both Blm10 mutant strains formed granules similar to wild-type indicating PSGs induced under glucose starvation form independently of Blm10. Scale bar represents 5 μm. *C*, indicated strains expressing α1-GFP were initially glucose starved for 24 h. Cells were collected and reinoculated in standard defined media containing glucose and monitored over a 15 min time course. α1-GFP readily formed granules in the *blm10Δ* strain and reentered the nucleus similar to the control strain. Scale bar represents 5 μm. *D*, glucose reintroduction as described in *C* for strains coexpressing GFP-Blm10 and α1-mCherry. Both GFP-Blm10 and α1-mCherry relocalized back to the nucleus within 15 min of glucose reintroduction. Scale bar represents 5 μm.

Blm10 was reported to be required for the granular cytosolic localization of CP following 5 days of growth in YPD or a change in media from glycerol to no carbon source (19, 55). Furthermore, induced overexpression of Blm10 using a galactose inducible system led to CP sequestration into cytosolic granules (19). We observed no change in CP localization upon overexpression of Blm10 using the GPD promoter in logarithmically growing cells maintained in the same carbon source (glucose). To test the impact of Blm10 overexpression on proteasome localization in cells completely deprived of carbon, we used a doubly tagged strain expressing GFP-Blm10 from a GPD promoter, and endogenously expressed α1-mCherry. Consistent with strains that contain only one tagged protein, Blm10 and the proteasome can be found in the nucleus of logarithmically growing cells. In sum, overexpression of Blm10 in this doubly tagged strain did not affect localization of CP or induce proteasome granules during logarithmic growth (Fig. 6*A*).

Following 24 h of glucose starvation, the proteasome can be found localized to granular structures in the cytoplasm. GFP-Blm10 did not colocalize with these granules, but instead remained nuclear (Fig. 6*A*). This suggests that unbound Blm10 does not have the signal for granule targeting. It was surprising that we did not see any Blm10 granules as previous reports of quiescence or a switch from glycerol to no carbon have shown colocalization of Blm10 and CP in PSGs (55, 62); however, we cannot exclude that the strong signal in the nucleus obscured the presence of weaker GFP-Blm10 granules. It should be noted that native gel analysis did show a reduction of Blm10 binding to CP under these conditions as we observed the appearance of RP_2_-CP and RP-CP complexes despite the presence of unbound Blm10 (Fig. S5*B*). Apparently, these conditions are less favorable for Blm10-CP interactions, and it is possible that only CP complexes without Blm10 form granules under these conditions.

To further investigate the potential of Blm10 to form granules upon glucose starvation, we tagged Blm10 with GFP under its endogenous promoter using a Cre-Lox-based approach. As expected, GFP-Blm10 localized to the nucleus along with α1-mCherry during logarithmic growth. Following glucose starvation, defined granules were observed for α1-mCherry while GFP-Blm10 appeared diffuse in the cytoplasm as well as in granule-like structures. While both α1-mCherry and GFP-Blm10 showed a change in localization from the nucleus to the cytoplasm, we observed no colocalization of signals (Fig. 6*A*). Consistent with the lack of colocalization, the granules that formed under our assay conditions did not depend on Blm10 as a *blm10Δ* strain was able to successfully form proteasome granules (Fig. 6*B*). As Blm10 has been reported to both colocalize with and aid in PSG formation during quiescence, we tested both our strain and the previously reported strain (generous gift from C. Enenkel) for granule formation following glucose starvation. It is possible that Blm10 may be important for granules that form under certain conditions of carbon limitation but not others.

Under the conditions used in our lab, strains expressing β5-GFP but lacking Blm10 readily formed granules similar to wild-type upon glucose starvation (Fig. 6*B*). It should be noted that we recently observed that tagging β5, one of the catalytic subunits of CP, resulted in impaired growth (particularly in combination with a deletion of RPN4). A growth phenotype was also observed for the β5-GFP *blm10Δ* strain (Fig. S2*A*). While our lab has not observed aberrant proteasome localization with β5-GFP-tagged strains under conditions of glucose starvation, we did observe β5-GFP-tagged proteasomes in granules upon nitrogen starvation in strains defective for autophagy. This was unique for β5-GFP and β4-GFP-tagged proteasomes as it was not observed with several other CP-tagged strains (β1, β2, α1, α6) suggesting that the GFP tag on β5 can alter the proteasome conformation, composition, or function (63). This may suggest that, depending on the physiological state of the cells, β5 GFP-tagged proteasomes may behave differently. Nevertheless, in our hands, Blm10 was not essential for the formation of PSGs upon glucose starvation in either β5- or α1- GFP tagged strains.

Proteasome storage granules dissipate and proteasomes relocalize to the nucleus upon glucose reintroduction to starved cells. Blm10 has been shown to be required for efficient and timely relocalization of CPs under this condition. In wild-type cells, PSGs dissipate within 15 min of nutrient reintroduction, but cells lacking Blm10 showed a delay of CP reentry of about 2 h (19). To test whether Blm10 played a role in CP reentry into the nucleus, we first induced granule formation by glucose starvation followed by glucose reintroduction. α1-GFP granules disappeared within 15 min upon glucose reintroduction with the majority of α1-GFP fluorescent signal relocalized to the nucleus (Fig. 6*C*). The absence of Blm10 delayed nuclear relocalization by about 5–10 min, indicating that the absence of Blm10 also impacts the efficiency of the process under our conditions. However, this was not nearly as dramatic as when glucose was added to quiescent cells. Thus, glucose starvation-induced PSGs can form, and proteasomes can reenter the nucleus, in the absence of Blm10 (Fig. 6*C*).

In light of these results, we next aimed to determine if Blm10 dynamics under glucose starvation were similar to proteasome CP dynamics. To test this, strains with endogenously expressed GFP-Blm10 and α1-mCherry were grown for 24 h in media lacking glucose. This resulted in Blm10 localization to the cytosol and the formation of granule-like structures. Five minutes after glucose reintroduction, the GFP-Blm10 fluorescence was less abundant in granules and fluorescence was observed in the nucleus and cytoplasm. After 15 min, GFP-Blm10 was enriched in the nucleus to similar levels as seen during logarithmic growth (Fig. 6*D*). Thus, Blm10 granules, like PSGs, are readily reversible and can disintegrate quickly following addition of glucose.

## Discussion

The CP houses the proteolytic active sites of the proteasome. However, the ability to degrade ubiquitinated and folded proteins is dependent upon the interaction of CP with RP. RP provides the receptors that recognize substrates, as well as contains the ATPase activity that unfolds and threads the substrates into the CP. Nevertheless, there are other regulators that can bind CP at the same surface as RP, such as human PI31, for which Fub1 seems to be the yeast ortholog, and Blm10 (PA200 in humans). How the cell controls or manages which regulator is bound to the CP is poorly understood. In addition to competitive binding, which is modulated by affinity and protein levels, CP–regulator interactions could be actively controlled. To determine the extent to which the association between Blm10 and CP is actively regulated, we altered the expression of this regulator and determined the proteasome landscape under those conditions. Interestingly, while overexpression of Blm10 resulted in a dramatic reduction of RP bound to mature CP, we only saw a modest impact with respect to immature CP.

### Blm10 and immature CP

Pba1-Pba2 is a heterodimeric assembly chaperone that guides the formation of the CP α-ring as it aids in the incorporation of α5 and α6. Furthermore, its association with the α-ring physically blocks the surface where RP would bind immature CP, thereby preventing RP from binding (18). As Blm10 binding to CP involves a pocket between α5 and α6 and takes up much of the α-ring surface, binding of Pba1-Pba2 and Blm10 is mutually exclusive. This suggests that Blm10 binds to immature CP that is assembled independent of Pba1-Pba2, Blm10 binds to immature CP after Pba1-Pba2, or Blm10 binds immature CP prior to Pba1-Pba2. While our data cannot distinguish between these forms, it is intriguing that strong overexpression of Blm10 can readily displace RP from CP, but not Pba1-Pba2 from immature CP (Fig. S1*A*). Pba1-Pba2 has been shown to bind immature CP with very high affinity due to specific conformational changes within immature CP upon binding to both Pba1-2 and Ump1 (13, 18, 34). Therefore, it seems that Blm10 works through an alternative pathway. Either Pba1-Pba2 is substoichiometric or a subset of immature CP must have a different conformation. Lack of Pba1-Pba2 in purifications using Ump1-flag, where Blm10 was found and only β7 was missing (36), *versus* the observed presence of Pba1-Pba2 and Blm10 in Ump1-TAP purifications where both β6 and β7 were missing, might indicate that Pba1-Pba2 is lost late in 1/2 CP maturation. However, it is likely some conformational changes have to occur to facilitate an exchange between Pba1-2 and Blm10. That said, the human Pba1-Pba2 orthologues, PAC1-PAC2, are proposed to remain bound to immature CP until it matures, suggesting Blm10 would not replace it (17). It remains to be determined if Blm10 binding to immature CP is structurally similar to its binding to mature CP. Regardless, it appears that, unlike what we observed for CP binding, the binding of Blm10 to immature CP does not involve a simple distribution based on affinities and protein levels.

### Blm10 binding to mature CP

Under normal physiological conditions, our data show that Blm10 is found solely bound to CP-containing complexes and not detectable as a free protein (see Fig. 2*B*). Consistent with this, increased levels of Blm10 seem to readily displace RP from CP to such an extent that free Blm10 only accumulates after the vast majority of CP has two Blm10 bound. That said, we do observe a small fraction of Blm10-CP-RP that remains present, but we are uncertain if this is a specific subpopulation (see Fig. 2*B*). Considering most CP and RP subunits are essential, we speculate that this amount of Blm10-CP-RP is the minimal amount of RP-CP required to perform the essential functions under normal growth conditions. Apparently, the low RP-CP levels in cells overexpressing *BLM10* are sufficient to support cell growth under optimal conditions (see Fig. 3*C*).

Supporting our notion that there is direct competition between Blm10 and RP, RP-CP only reappeared in conditions that reduced the levels of free Blm10. Furthermore, the overexpression of *Blm10Δ3* showed no ability to reduce RP-CP levels in the cell, indicating that reduction of RP-CP is indeed due to direct competition between RP and Blm10. An important implication of this observed competition is that changes in Blm10 levels have a direct impact on RP-CP levels in the cell. Consistent with this, endogenous levels of Blm10 in the cell are normally substoichiometric allowing for an abundance of 26S (RP-CP complexes).

### Blm10 stoichiometry and function

Blm10 is normally present at levels much lower than CP, which suggests that Blm10 either acts only on a subset of CP or functions in a temporal and reusable fashion on all CP. The former is consistent with a function in the degradation of certain substrates, such as acetylated histones or tau-441 (30, 33). Here, depending on a cell’s need, only a subset of Blm10-CP complexes would be required. For substrates to enter the CP, they need to be recognized and unfolded. It remains unclear how Blm10-CP would recognize these substrates. Blm10 was reported to have a bromodomain-like region that can recognize acetylated substrates; however, recent structures raised some questions concerning this recognition (42, 43). As Blm10 lacks ATPase activity, its substrates must already be (partly) unfolded to allow for entry into CP (30, 31, 40). This would suggest that Blm10-specific substrates would either be intrinsically disordered proteins or Blm10 requires the assistance of an unfolding ATPase. The ATPase p97/Cdc48 is known to assist the 26S proteasome in degradation of tail-lagging substrates (73, 74). Alternatively, there might be a role for hybrid proteasomes (Blm10-CP-RP) in degrading substrates. Hybrid complexes can be readily observed under normal conditions and the RP can bind and unfold ubiquitinated substrates. Since Blm10 would bind on the other end of CP, its role could either be as an allosteric regulator or it could impact degradation by regulating peptide release (30, 42, 43). Although, one study looking at cellular peptides in yeast did not observe a major difference resulting from *BLM10* deletion (75).

Consistent with a role in a temporal, reusable fashion, Blm10 has been suggested to act as a chaperone in CP assembly due to its ability to bind immature CP (35, 36, 57). However, to function effectively as a chaperone, one would expect the protein to be, to some extent, present in a free form that could readily associate with newly formed complexes. It remains unclear how Blm10 would function in this capacity compared with Pba1-Pba2. Alternatively, Blm10 could be reusable as a factor that facilitates CP cellular localization as has been observed during quiescence and carbon starvation (19, 55). A function for shuttling of CP into PSGs has been proposed; however, these studies indicate that Blm10 colocalizes with the CP in these granules. Considering the low endogenous levels of Blm10 relative to CP and the lack of apparent upregulation under stress conditions (such as prolonged growth in YPD and glucose starvation), it would imply that only a small fraction of CPs could be targeted to proteasome storage granules by Blm10. If Blm10 were responsible for targeting all CPs to granules, it should be upregulated to levels where one Blm10 could bind each CP or at least utilize a mechanism where Blm10 could bind and unbind CPs to shuttle them to these granules.

### Autophagic degradation of Blm10

Nitrogen starvation and proteasome inhibitor treatment both induce autophagy of proteasomes (RP and CP complexes). However, it is less clear what happens to other proteasome regulators. Here, we show that nitrogen starvation also induces autophagy of Blm10-CP complexes as well as unbound Blm10. The latter is consistent with a previous report where Blm10-GFP was monitored following nitrogen starvation (55). Since both Blm10 and RP-CP complexes are degraded, the reduction of Blm10 in the overexpressing strain causes only minute recovery of RP-CP complexes upon nitrogen starvation (Fig. 4*B*). When cells were grown in YPD for 24–48 h or starved of glucose, there was no reported autophagy of proteasomes. Therefore, it was particularly surprising to observe that Blm10-CP complexes and unbound Blm10 were specifically targeted for autophagic degradation under these conditions. Indeed, cells overexpressing Blm10 showed a strong recovery of RP-CP complexes because Blm10 was degraded *via* autophagy. Considering we observed a reduction in GFP-Blm10-specific smearing on native gel in a strain that is defective in ubiquitination at a nonpermissive temperature (*uba1-ts*), this was likely triggered by Blm10 ubiquitination. While a deletion of *RPN4* indicated that reformation of RP-CP complexes following stress is, in part, due to syntheses of new complexes, our data also suggest that autophagic degradation of ubiquitinated free Blm10 could cause release of CP from Blm10 to allow for the formation of RP-CP complexes.

These observed effects were more dramatic upon Blm10 overexpression; however, we also observed autophagic degradation of endogenously expressed Blm10, indicating these observations are physiologically relevant. While we report a strong stabilization of Blm10-CP complexes in strains defective for autophagy, we also observed some modest effect of treatment with proteasome inhibitor, in particular, for unbound *Blm10Δ3*. As this degradation was minor compared with the autophagy, we expect this to result from general misfolded and protein quality-control-based pathways rather than a specific cellular response or regulation of proteasome complexes. The cell’s ability to degrade unbound Blm10 and CP-bound Blm10, but not other forms of CP, could indicate that Blm10 possesses a specific autophagy targeting signal although specific modifications have yet to be identified. Alternatively, the binding of Blm10 to the CP might induce conformational changes within this complex that make it more readily recognized and targeted for degradation compared with CP alone or RP-CP complexes. The human ortholog of Blm10, PA200, has indeed been shown to induce conformational changes in CP as binding changes the specificity of the proteolytic active sites (42). While the specific mechanism of how Blm10-bound complexes are targeted for degradation remains to be determined, it is clear that they are degraded through the process of autophagy.

### Blm10 and nuclear import

Under optimal growth conditions, Blm10 is highly enriched in the nucleus, but it has been observed to be required for formation of cytosolic granules in quiescence as well as cells grown in and depleted of glycerol (19, 55). In our hands, endogenously expressed Blm10 was able to form granule-like structures under glucose starvation similar to what has been reported for cells starved of glycerol (55); however, most of these structures were devoid of CP. Furthermore, Blm10 is important for efficient nuclear import of CP upon removal of nutrient stress (Fig. 6*C* and (19)). While we observed a role for Blm10 in proteasome nuclear import after glucose starvation, this function was not as essential as has been reported for recovery from quiescence.

In all, our data show that Blm10 can directly compete with RP for binding to CP, suggesting Blm10 levels may be tightly regulated. As formation of 26S proteasomes is vital to cell survival, it’s important to understand that any changes in levels of potential competitors such as Blm10 can have effects on 26S proteasome levels. As such, it appears that cells utilize a mechanism of clearing potential competitors as prolonged growth in YPD (24–48 h) results in the selective, autophagic degradation of Blm10 leading to reduced levels of Blm10-CP complexes and increased RP-CP levels. It will be important to understand the mechanisms of how these various complexes are differentially modified in response to specific stress conditions.

## Experimental procedures

### Strains

All yeast strains used in this study are listed in Table S1. Gene disruptions or the introduction of tags at the endogenous locus was achieved using standard PCR-based approaches (44, 76). To keep the *BLM10* endogenous promoter, we utilized a Cre-Lox-based approach to tag Blm10 with Venus (77). For replacing the endogenous promoter and concomitantly tagging Blm10 with eGFP, we used a PCR-based approach to insert the promoters from the CYC1, ADH, or GPD genes (Table S2). MAHQ1 was a generous gift from Dr Matouschek (70).

### Yeast growth conditions

Yeast strains were grown in YPD media at 30 °C unless stated otherwise. To induce starvation, overnight cultures were inoculated in fresh YPD at an OD_600_ of 0.5. Cells were grown to an OD_600_ between 1 and 1.5 (∼4 h). Cells were collected by centrifugation, washed with sterile water, and resuspended in SD complete media lacking indicated nutrients (starvation media) to a final OD_600_ of 1.5. Media used for glucose starvation contained 0.17% yeast nitrogen base with 0.5% (NH_4_)_2_SO_4_ supplemented with 1× amino acid mix, 1× uracil, and 1× adenine. Nitrogen starvation media contained 0.17% yeast nitrogen base (without (NH_4_)_2_SO_4_) and 2% dextrose. Log phase and starvation-induced cells were grown either at 30 °C or 37 °C with constant shaking. Samples for specific timepoints were harvested by centrifugation, frozen dropwise in liquid nitrogen, and stored at –80 °C for further processing (78).

### Phenotype screen

Yeast cells were grown in YPD to an OD_600_ of 1. Cells from 1 ml of culture were collected by centrifugation (17,000*g* for 1 min) and washed using sterile water. Cell pellets were resuspended in sterile water and fourfold serial dilutions were performed for each sample using a 96-well plate. Using a pin array, a droplet of each dilution of cells was spotted onto YPD plates. Plates were incubated at either 30 °C or 37 °C for 1–3 days.

### Western blot analysis

Depending on the purpose of the assay, cell lysates were made using different methods as indicated in figure legends. Cell lysis by grinding in N_2_ (l) was achieved as described previously (78). In brief, cell pellets at –80 °C were transferred to prechilled mortars and ground with a pestle to powder in the presence of liquid nitrogen. The powder was transferred to an Eppendorf tube, resuspended in lysis buffer (50 mM Tris-HCl [pH 7.5], 5 mM MgCl_2_, 1 mM ATP, 1 mM EDTA) and incubated on ice for 10 min. Next, lysates were cleared by centrifugation in a microfuge at 17,000*g* for 2 min at 4 °C. Supernatants were collected, and protein concentrations were measured using the NanoDrop. Equal amounts of protein for each sample were loaded for both SDS-PAGE and native gel electrophoresis. For alkaline cell lysis, two ODs of cells were frozen and then resuspended in 100 μl water followed by the addition of 100 μl 200 mM NaOH. Samples were incubated at room temperature for 5 min and collected by centrifugation in a microcentrifuge for 2 min at 17,000*g*. Following aspiration, pellets were resuspended in 50 μl alkaline lysis buffer (60 mM Tris-HCl [pH 6.8], 5% glycerol, 2% SDS, 4% β-mercaptoethanol, 0.0025% bromophenol blue). Samples were boiled at 96 °C for 3 min and cleared by centrifugation at 17,000*g*. In total, 6 μl of supernatant was loaded for SDS-PAGE. After separation of the lysate, gels were transferred to PVDF membranes and immunoblotted for proteins of interest. Primary antibodies used for immunoblotting were against GFP (1:500; Roche, #11814460001), Blm10 (1:1000; Enzo Life Sciences #XO8100), Rpn8 (1:10,000; #4797, generous gift from Dan Finley, Harvard Medical School, Boston, MA), Pba1 and Pba2 (1:500; (18)), α7 (1:1000; Enzo Life Sciences, #02081203), and Pgk1 (1:4000; Invitrogen, #459250). Secondary antibodies conjugated to horseradish peroxidase were purchased from Rockland Immunochemicals. Peroxidase activity was visualized using the Immobilon Forte Western HRP substrate from Millipore. Immunoblot images of luminescence were captured using a Syngene G-box imager from Syngene with GeneSnap software. Pgk1 was utilized for a loading control.

### Fluorescence microscopy

Imaging live yeast cells expressing GFP, Venus, or mCherry fusion proteins was conducted as described previously (79). In brief, the equivalent of two ODs of cells were spun down and resuspended in 20 μl of the culture media. In total, 3 μl of this cell suspension was sandwiched between an agarose-padded slide and coverslip. The agarose-padded slides were prepared by resuspending agarose to a 1% final concentration in SD complete media (0.17% yeast nitrogen base containing 0.5% (NH_4_)_2_SO_4_, amino acids, uracil, adenine, and 2% dextrose) by heating the solution to 96 °C. Next, 30 μl of the agarose solution was spread out onto the slide by placing a second slide on top of the droplet. Once solidified, the top slide was removed leaving the agar pad upon the original slide. Images were acquired using fluorescence microscopy on a Nikon Eclipse TE2000-S microscope at room temperature using 600× magnification (Plan Apo 60x/1.40 objective) and a Retiga R3^tm^ camera from QImaging. Images were collected using the Metamorph software (Molecular Devices) and analyzed with ImageJ.

### Native gel electrophoresis and activity assay

Native gel analyses optimized for proteasome complexes were done to determine the composition/distribution of proteasome complexes in the cell. For each sample, 300 μg of cell lysate prepared by cryo-grinding was loaded on native gel. Electrophoresis, using a 3.5% acrylamide gel, was performed at 96 V for 2.5 h at 4 °C to separate the protein complexes. Gels were imaged to visualize fluorescently tagged proteins using a Typhoon 9410 imager. Fluorescent tags were visualized using the following excitations and filters: GFP (488 nm and 526SP) and Venus (532 nm and 526SP). Next, to visualize bands with proteasome activity, an in-gel LLVY-AMC hydrolysis (activity) assay was performed as previously described (78). Images were acquired using a G-Box imaging system from SynGene and GeneSnap software.

### Proteasome purification

Overnight cultures in YPD were used to inoculate 4.5 L of fresh YPD to a final OD_600_ of 0.5. Cells were allowed to grow for 6–8 h before harvesting by centrifugation to produce a large pellet of logarithmically growing cells. Cells were washed with water before being resuspended in lysis buffer (1.5 pellet volumes: 50 mM Tris [pH 8.0], 5 mM MgCl_2_, 1 mM EDTA, 1 mM ATP supplemented with ProBlock protease inhibitor cocktail from GoldBio). Cells were lysed using a French Press at a pressure of 20,000 internal cell psi. To clear lysates, samples were centrifuged at 11,000 rpm in a Beckman Coulter Aventi J-E centrifuge (rotor: JA-17) for 20 min, and the resulting supernatant was filtered through a cheesecloth. The cleared lysate was incubated with Antigen Affinity Gel Rabbit IgG resin (whole molecule from MP Biomedicals, LLC) for 1 h at 4 °C with constant rotation (750 μl of IgG slurry for 1.5 L of culture). Resin was collected using a Biorad econo-column (0.5 ml wide) and washed with 50 bed volumes of ice-cold Buffer 2 (50 mM Tris [pH 7.5], 5 mM MgCl_2_, 1 mM EDTA, 1 mM ATP, and 20 mM NaCl). Next, resin was washed with 15 bed volumes ice-cold elution buffer (50 mM Tris [pH 7.5], 5 mM MgCl_2_, 1 mM EDTA, 1 mM DTT, 1 mM ATP). Proteasome complexes were eluted by an incubation with 750 μl elution buffer containing 8 μl GST-Tev protease (stock concentration: 1.519 mg/ml) for 1 h at 30 °C. Collected eluate was incubated with constant rotation for 20 min at 4 °C with glutathione resin to remove Tev protease. After removal of the resin using centrifugation and a spin column, proteasomes were concentrated using a concentrator with 100 kDa MW cutoff (PALL Life Sciences). Concentrated samples were analyzed by SDS-PAGE and native gel electrophoresis as described earlier.

## Data availability

All data are contained within the article or the Supporting information.

## Supporting information

This article contains supporting information (70).

## Conflict of interest

The authors declare that they have no conflicts of interest with the contents of this article.

## References

[1] Groll M., Ditzel L., Lowe J., Stock D., Bochtler M., Bartunik H.D., Huber R. (1997). Structure of 20S proteasome from yeast at 2.4 A resolution. Nature.

[2] Marques A.J., Palanimurugan R., Matias A.C., Ramos P.C., Dohmen R.J. (2009). Catalytic mechanism and assembly of the proteasome. Chem. Rev..

[3] Reits E., Griekspoor A., Neijssen J., Groothuis T., Jalink K., van Veelen P., Janssen H., Calafat J., Drijfhout J.W., Neefjes J. (2003). Peptide diffusion, protection, and degradation in nuclear and cytoplasmic compartments before antigen presentation by MHC class I. Immunity.

[4] Vigneron N., Van den Eynde B.J. (2014). Proteasome subtypes and regulators in the processing of antigenic peptides presented by class I molecules of the major histocompatibility complex. Biomolecules.

[5] Groll M., Bajorek M., Kohler A., Moroder L., Rubin D.M., Huber R., Glickman M.H., Finley D. (2000). A gated channel into the proteasome core particle. Nat. Struct. Biol..

[6] Deveraux Q., Ustrell V., Pickart C., Rechsteiner M. (1994). A 26 S protease subunit that binds ubiquitin conjugates. J. Biol. Chem..

[7] Husnjak K., Elsasser S., Zhang N.X., Chen X., Randles L., Shi Y., Hofmann K., Walters K.J., Finley D., Dikic I. (2008). Proteasome subunit Rpn13 is a novel ubiquitin receptor. Nature.

[8] Schreiner P., Chen X., Husnjak K., Randles L., Zhang N., Elsasser S., Finley D., Dikic I., Walters K.J., Groll M. (2008). Ubiquitin docking at the proteasome through a novel pleckstrin-homology domain interaction. Nature.

[9] Shi Y., Chen X., Elsasser S., Stocks B.B., Tian G., Lee B.H., Shi Y., Zhang N., de Poot S.A., Tuebing F., Sun S., Vannoy J., Tarasov S.G., Engen J.R., Finley D. (2016). Rpn1 provides adjacent receptor sites for substrate binding and deubiquitination by the proteasome. Science.

[10] Rabl J., Smith D.M., Yu Y., Chang S.C., Goldberg A.L., Cheng Y. (2008). Mechanism of gate opening in the 20S proteasome by the proteasomal ATPases. Mol. Cell.

[11] Sadre-Bazzaz K., Whitby F.G., Robinson H., Formosa T., Hill C.P. (2010). Structure of a Blm10 complex reveals common mechanisms for proteasome binding and gate opening. Mol. Cell.

[12] Smith D.M., Chang S.C., Park S., Finley D., Cheng Y., Goldberg A.L. (2007). Docking of the proteasomal ATPases' carboxyl termini in the 20S proteasome's alpha ring opens the gate for substrate entry. Mol. Cell.

[13] Stadtmueller B.M., Kish-Trier E., Ferrell K., Petersen C.N., Robinson H., Myszka D.G., Eckert D.M., Formosa T., Hill C.P. (2012). Structure of a proteasome Pba1-Pba2 complex: Implications for proteasome assembly, activation, and biological function. J. Biol. Chem..

[14] Strehl B., Seifert U., Kruger E., Heink S., Kuckelkorn U., Kloetzel P.M. (2005). Interferon-gamma, the functional plasticity of the ubiquitin-proteasome system, and MHC class I antigen processing. Immunol. Rev..

[15] McCutchen-Maloney S.L., Matsuda K., Shimbara N., Binns D.D., Tanaka K., Slaughter C.A., DeMartino G.N. (2000). cDNA cloning, expression, and functional characterization of PI31, a proline-rich inhibitor of the proteasome. J. Biol. Chem..

[16] Li X., Thompson D., Kumar B., DeMartino G.N. (2014). Molecular and cellular roles of PI31 (PSMF1) protein in regulation of proteasome function. J. Biol. Chem..

[17] Hirano Y., Hendil K.B., Yashiroda H., Iemura S., Nagane R., Hioki Y., Natsume T., Tanaka K., Murata S. (2005). A heterodimeric complex that promotes the assembly of mammalian 20S proteasomes. Nature.

[18] Wani P.S., Rowland M.A., Ondracek A., Deeds E.J., Roelofs J. (2015). Maturation of the proteasome core particle induces an affinity switch that controls regulatory particle association. Nat. Commun..

[19] Weberruss M.H., Savulescu A.F., Jando J., Bissinger T., Harel A., Glickman M.H., Enenkel C. (2013). Blm10 facilitates nuclear import of proteasome core particles. EMBO J..

[20] Chen X., Barton L.F., Chi Y., Clurman B.E., Roberts J.M. (2007). Ubiquitin-independent degradation of cell-cycle inhibitors by the REGgamma proteasome. Mol. Cell.

[21] Li X., Amazit L., Long W., Lonard D.M., Monaco J.J., O'Malley B.W. (2007). Ubiquitin- and ATP-independent proteolytic turnover of p21 by the REGgamma-proteasome pathway. Mol. Cell.

[22] Li X., Lonard D.M., Jung S.Y., Malovannaya A., Feng Q., Qin J., Tsai S.Y., Tsai M.J., O'Malley B.W. (2006). The SRC-3/AIB1 coactivator is degraded in a ubiquitin- and ATP-independent manner by the REGgamma proteasome. Cell.

[23] Nie J., Wu M., Wang J., Xing G., He F., Zhang L. (2010). REGgamma proteasome mediates degradation of the ubiquitin ligase Smurf1. FEBS Lett..

[24] Suzuki R., Moriishi K., Fukuda K., Shirakura M., Ishii K., Shoji I., Wakita T., Miyamura T., Matsuura Y., Suzuki T. (2009). Proteasomal turnover of hepatitis C virus core protein is regulated by two distinct mechanisms: A ubiquitin-dependent mechanism and a ubiquitin-independent but PA28gamma-dependent mechanism. J. Virol..

[25] Hendil K.B., Khan S., Tanaka K. (1998). Simultaneous binding of PA28 and PA700 activators to 20 S proteasomes. Biochem. J..

[26] Hoffman L., Pratt G., Rechsteiner M. (1992). Multiple forms of the 20 S multicatalytic and the 26 S ubiquitin/ATP-dependent proteases from rabbit reticulocyte lysate. J. Biol. Chem..

[27] Tanahashi N., Murakami Y., Minami Y., Shimbara N., Hendil K.B., Tanaka K. (2000). Hybrid proteasomes. Induction by interferon-gamma and contribution to ATP-dependent proteolysis. J. Biol. Chem..

[28] Forster A., Masters E.I., Whitby F.G., Robinson H., Hill C.P. (2005). The 1.9 A structure of a proteasome-11S activator complex and implications for proteasome-PAN/PA700 interactions. Mol. Cell.

[29] Zhang Z., Clawson A., Realini C., Jensen C.C., Knowlton J.R., Hill C.P., Rechsteiner M. (1998). Identification of an activation region in the proteasome activator REGalpha. Proc. Natl. Acad. Sci. U. S. A..

[30] Dange T., Smith D., Noy T., Rommel P.C., Jurzitza L., Cordero R.J., Legendre A., Finley D., Goldberg A.L., Schmidt M. (2011). Blm10 protein promotes proteasomal substrate turnover by an active gating mechanism. J. Biol. Chem..

[31] Lopez A.D., Tar K., Krugel U., Dange T., Ros I.G., Schmidt M. (2011). Proteasomal degradation of Sfp1 contributes to the repression of ribosome biogenesis during starvation and is mediated by the proteasome activator Blm10. Mol. Biol. Cell.

[32] Mandemaker I.K., Geijer M.E., Kik I., Bezstarosti K., Rijkers E., Raams A., Janssens R.C., Lans H., Hoeijmakers J.H., Demmers J.A., Vermeulen W., Marteijn J.A. (2018). DNA damage-induced replication stress results in PA200-proteasome-mediated degradation of acetylated histones. EMBO Rep..

[33] Qian M.X., Pang Y., Liu C.H., Haratake K., Du B.Y., Ji D.Y., Wang G.F., Zhu Q.Q., Song W., Yu Y., Zhang X.X., Huang H.T., Miao S., Chen L.B., Zhang Z.H. (2013). Acetylation-mediated proteasomal degradation of core histones during DNA repair and spermatogenesis. Cell.

[34] Kock M., Nunes M.M., Hemann M., Kube S., Dohmen R.J., Herzog F., Ramos P.C., Wendler P. (2015). Proteasome assembly from 15S precursors involves major conformational changes and recycling of the Pba1-Pba2 chaperone. Nat. Commun..

[35] Fehlker M., Wendler P., Lehmann A., Enenkel C. (2003). Blm3 is part of nascent proteasomes and is involved in a late stage of nuclear proteasome assembly. EMBO Rep..

[36] Marques A.J., Glanemann C., Ramos P.C., Dohmen R.J. (2007). The C-terminal extension of the beta7 subunit and activator complexes stabilize nascent 20 S proteasomes and promote their maturation. J. Biol. Chem..

[37] Blickwedehl J., Agarwal M., Seong C., Pandita R.K., Melendy T., Sung P., Pandita T.K., Bangia N. (2008). Role for proteasome activator PA200 and postglutamyl proteasome activity in genomic stability. Proc. Natl. Acad. Sci. U. S. A..

[38] Blickwedehl J., McEvoy S., Wong I., Kousis P., Clements J., Elliott R., Cresswell P., Liang P., Bangia N. (2007). Proteasomes and proteasome activator 200 kDa (PA200) accumulate on chromatin in response to ionizing radiation. Radiat. Res..

[39] Khor B., Bredemeyer A.L., Huang C.Y., Turnbull I.R., Evans R., Maggi L.B., White J.M., Walker L.M., Carnes K., Hess R.A., Sleckman B.P. (2006). Proteasome activator PA200 is required for normal spermatogenesis. Mol. Cell Biol..

[40] Tar K., Dange T., Yang C., Yao Y., Bulteau A.L., Salcedo E.F., Braigen S., Bouillaud F., Finley D., Schmidt M. (2014). Proteasomes associated with the Blm10 activator protein antagonize mitochondrial fission through degradation of the fission protein Dnm1. J. Biol. Chem..

[41] Ustrell V., Hoffman L., Pratt G., Rechsteiner M. (2002). PA200, a nuclear proteasome activator involved in DNA repair. EMBO J..

[42] Toste Rego A., da Fonseca P.C.A. (2019). Characterization of fully recombinant human 20S and 20S-PA200 proteasome complexes. Mol. Cell.

[43] Guan H., Wang Y., Yu T., Huang Y., Li M., Saeed A., Perculija V., Li D., Xiao J., Wang D., Zhu P., Ouyang S. (2020). Cryo-EM structures of the human PA200 and PA200-20S complex reveal regulation of proteasome gate opening and two PA200 apertures. PLoS Biol..

[44] Goldstein A.L., McCusker J.H. (1999). Three new dominant drug resistance cassettes for gene disruption in Saccharomyces cerevisiae. Yeast.

[45] Cascio P., Call M., Petre B.M., Walz T., Goldberg A.L. (2002). Properties of the hybrid form of the 26S proteasome containing both 19S and PA28 complexes. EMBO J..

[46] Kopp F., Dahlmann B., Kuehn L. (2001). Reconstitution of hybrid proteasomes from purified PA700-20 S complexes and PA28alphabeta activator: Ultrastructure and peptidase activities. J. Mol. Biol..

[47] De La Mota-Peynado A., Lee S.Y., Pierce B.M., Wani P., Singh C.R., Roelofs J. (2013). The proteasome-associated protein Ecm29 inhibits proteasomal ATPase activity and *in vivo* protein degradation by the proteasome. J. Biol. Chem..

[48] Leggett D.S., Hanna J., Borodovsky A., Crosas B., Schmidt M., Baker R.T., Walz T., Ploegh H., Finley D. (2002). Multiple associated proteins regulate proteasome structure and function. Mol. Cell.

[49] Schmidt M., Haas W., Crosas B., Santamaria P.G., Gygi S.P., Walz T., Finley D. (2005). The HEAT repeat protein Blm10 regulates the yeast proteasome by capping the core particle. Nat. Struct. Mol. Biol..

[50] Kleijnen M.F., Roelofs J., Park S., Hathaway N.A., Glickman M., King R.W., Finley D. (2007). Stability of the proteasome can be regulated allosterically through engagement of its proteolytic active sites. Nat. Struct. Mol. Biol..

[51] Lee S.Y., De la Mota-Peynado A., Roelofs J. (2011). Loss of Rpt5 protein interactions with the core particle and Nas2 protein causes the formation of faulty proteasomes that are inhibited by Ecm29 protein. J. Biol. Chem..

[52] Marshall R.S., Li F., Gemperline D.C., Book A.J., Vierstra R.D. (2015). Autophagic degradation of the 26S proteasome is mediated by the dual ATG8/ubiquitin receptor RPN10 in arabidopsis. Mol. Cell.

[53] Waite K.A., De-La Mota-Peynado A., Vontz G., Roelofs J. (2016). Starvation induces proteasome autophagy with different pathways for core and regulatory particles. J. Biol. Chem..

[54] Klionsky D.J., Abdelmohsen K., Abe A., Abedin M.J., Abeliovich H., Acevedo Arozena A., Adachi H., Adams C.M., Adams P.D., Adeli K., Adhihetty P.J., Adler S.G., Agam G., Agarwal R., Aghi M.K. (2016). Guidelines for the use and interpretation of assays for monitoring autophagy (3rd edition). Autophagy.

[55] Marshall R.S., Vierstra R.D. (2018). Proteasome storage granules protect proteasomes from autophagic degradation upon carbon starvation. Elife.

[56] Janke C., Magiera M.M., Rathfelder N., Taxis C., Reber S., Maekawa H., Moreno-Borchart A., Doenges G., Schwob E., Schiebel E., Knop M. (2004). A versatile toolbox for PCR-based tagging of yeast genes: New fluorescent proteins, more markers and promoter substitution cassettes. Yeast.

[57] Li X., Kusmierczyk A.R., Wong P., Emili A., Hochstrasser M. (2007). beta-Subunit appendages promote 20S proteasome assembly by overcoming an Ump1-dependent checkpoint. EMBO J..

[58] Xie Y., Varshavsky A. (2001). RPN4 is a ligand, substrate, and transcriptional regulator of the 26S proteasome: A negative feedback circuit. Proc. Natl. Acad. Sci. U. S. A..

[59] Shirozu R., Yashiroda H., Murata S. (2015). Identification of minimum Rpn4-responsive elements in genes related to proteasome functions. FEBS Lett..

[60] Fleming J.A., Lightcap E.S., Sadis S., Thoroddsen V., Bulawa C.E., Blackman R.K. (2002). Complementary whole-genome technologies reveal the cellular response to proteasome inhibition by PS-341. Proc. Natl. Acad. Sci. U. S. A..

[61] Schmidt R.M., Schessner J.P., Borner G.H., Schuck S. (2019). The proteasome biogenesis regulator Rpn4 cooperates with the unfolded protein response to promote ER stress resistance. Elife.

[62] Gu Z.C., Wu E., Sailer C., Jando J., Styles E., Eisenkolb I., Kuschel M., Bitschar K., Wang X., Huang L., Vissa A., Yip C.M., Yedidi R.S., Friesen H., Enenkel C. (2017). Ubiquitin orchestrates proteasome dynamics between proliferation and quiescence in yeast. Mol. Biol. Cell.

[63] Waite K.A., Burris A., Roelofs J. (2020). Tagging the proteasome active site beta5 causes tag specific phenotypes in yeast. Sci. Rep..

[64] Lehmann A., Niewienda A., Jechow K., Janek K., Enenkel C. (2010). Ecm29 fulfils quality control functions in proteasome assembly. Mol. Cell.

[65] Mannhaupt G., Schnall R., Karpov V., Vetter I., Feldmann H. (1999). Rpn4p acts as a transcription factor by binding to PACE, a nonamer box found upstream of 26S proteasomal and other genes in yeast. FEBS Lett..

[66] Park S., Kim W., Tian G., Gygi S.P., Finley D. (2011). Structural defects in the regulatory particle-core particle interface of the proteasome induce a novel proteasome stress response. J. Biol. Chem..

[67] Wani P.S., Suppahia A., Capalla X., Ondracek A., Roelofs J. (2016). Phosphorylation of the C-terminal tail of proteasome subunit alpha7 is required for binding of the proteasome quality control factor Ecm29. Sci. Rep..

[68] Nemec A.A., Howell L.A., Peterson A.K., Murray M.A., Tomko R.J. (2017). Autophagic clearance of proteasomes in yeast requires the conserved sorting nexin Snx4. J. Biol. Chem..

[69] Reggiori F., Klionsky D.J. (2013). Autophagic processes in yeast: Mechanism, machinery and regulation. Genetics.

[70] Yu H., Singh Gautam A.K., Wilmington S.R., Wylie D., Martinez-Fonts K., Kago G., Warburton M., Chavali S., Inobe T., Finkelstein I.J., Babu M.M., Matouschek A. (2016). Conserved sequence preferences contribute to substrate recognition by the proteasome. J. Biol. Chem..

[71] Ghaboosi N., Deshaies R.J. (2007). A conditional yeast E1 mutant blocks the ubiquitin-proteasome pathway and reveals a role for ubiquitin conjugates in targeting Rad23 to the proteasome. Mol. Biol. Cell.

[72] Laporte D., Salin B., Daignan-Fornier B., Sagot I. (2008). Reversible cytoplasmic localization of the proteasome in quiescent yeast cells. J. Cell Biol.

[73] Beskow A., Grimberg K.B., Bott L.C., Salomons F.A., Dantuma N.P., Young P. (2009). A conserved unfoldase activity for the p97 AAA-ATPase in proteasomal degradation. J. Mol. Biol..

[74] Olszewski M.M., Williams C., Dong K.C., Martin A. (2019). The Cdc48 unfoldase prepares well-folded protein substrates for degradation by the 26S proteasome. Commun. Biol..

[75] Dasgupta S., Yang C., Castro L.M., Tashima A.K., Ferro E.S., Moir R.D., Willis I.M., Fricker L.D. (2016). Analysis of the yeast peptidome and comparison with the human peptidome. PLoS One.

[76] Longtine M.S., McKenzie A., Demarini D.J., Shah N.G., Wach A., Brachat A., Philippsen P., Pringle J.R. (1998). Additional modules for versatile and economical PCR-based gene deletion and modification in Saccharomyces cerevisiae. Yeast.

[77] Prein B., Natter K., Kohlwein S.D. (2000). A novel strategy for constructing N-terminal chromosomal fusions to green fluorescent protein in the yeast Saccharomyces cerevisiae. FEBS Lett..

[78] Roelofs J., Suppahia A., Waite K.A., Park S. (2018). Native gel approaches in studying proteasome assembly and chaperones. Methods Mol. Biol..

[79] Sundin B.A., Chiu C.H., Riffle M., Davis T.N., Muller E.G. (2004). Localization of proteins that are coordinately expressed with Cln2 during the cell cycle. Yeast.

