# Supplementary material for: Proteasome activator Blm10 levels and autophagic degradation directly impact the proteasome landscape
Source: J Biol Chem. 2021 Feb 25;296:100468. doi: 10.1016/j.jbc.2021.100468 (PMC8039559; doi:10.1016/j.jbc.2021.100468)
Supplement: Supplementary file 1 — Figures S1 to S5 and Tables S1 and S2 [file mmc1.pdf]

Supporting Information

Proteasome activator Blm10 levels and autophagic degradation directly impact the proteasome landscape

Alicia Burris[1,2], Kenrick A. Waite[1], Zach Reuter[2], Sam Ockerhausen[2], and Jeroen Roelofs[1,2#]

[1] Department of Biochemistry and Molecular Biology, University of Kansas Medical Center, Kansas City, 3901 Rainbow Blvd, HLSIC 1077, Kansas, USA

[2] Molecular, Cellular, and Developmental Biology Program, Division of Biology, Kansas State University, 338 Ackert Hall, Manhattan, Kansas 66506 USA

Contact

# Correspondence: jroelofs@kumc.edu

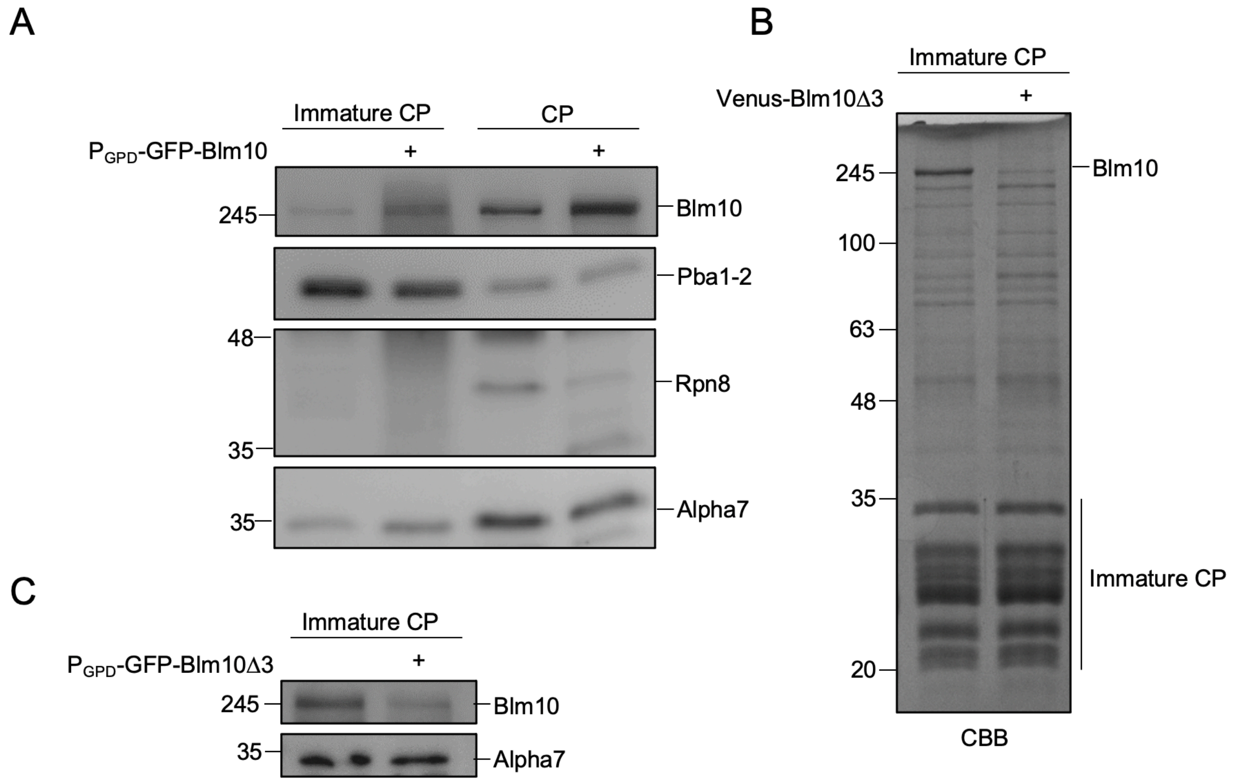

Supplementary Figure 1 - Blm10 fails to outcompete Pba1-2 as it does RP

(a) β4 ProtA-tagged strains were used to purify mature proteasomes while Ump1 ProtA-tagged strains allowed for purification of immature CP. Purified complexes were resolved using SDS-PAGE to determine the extent to which Blm10 could compete with RP and Pba1-2 for binding to mature and immature CP, respectively. Competition was determined through overexpression of Blm10. Immunoblotting for Blm10 allowed determination of the amount of Blm10 bound and co-purified with mature and immature CP. α7 was used as a loading control while antibodies against Pba1-2 and Rpn8, a subunit of RP, were used to determine the extent of Blm10 competition (b) Immature CP was purified from strains expressing endogenous levels of Blm10Δ3. Samples were separated using SDS-PAGE followed by staining with CBB. The mutant form of Blm10 showed reduced binding to immature CP similarly to mature CP. (c) To determine whether overexpression of Blm10 could force binding to immature CP, Ump1-ProtA tagged strains overexpressing Blm10 were used to again purify immature CP complexes. Immunoblots against Blm10 were utilized to determine the extent of Blm10 binding and subsequent purification. α7 was used as a loading control.

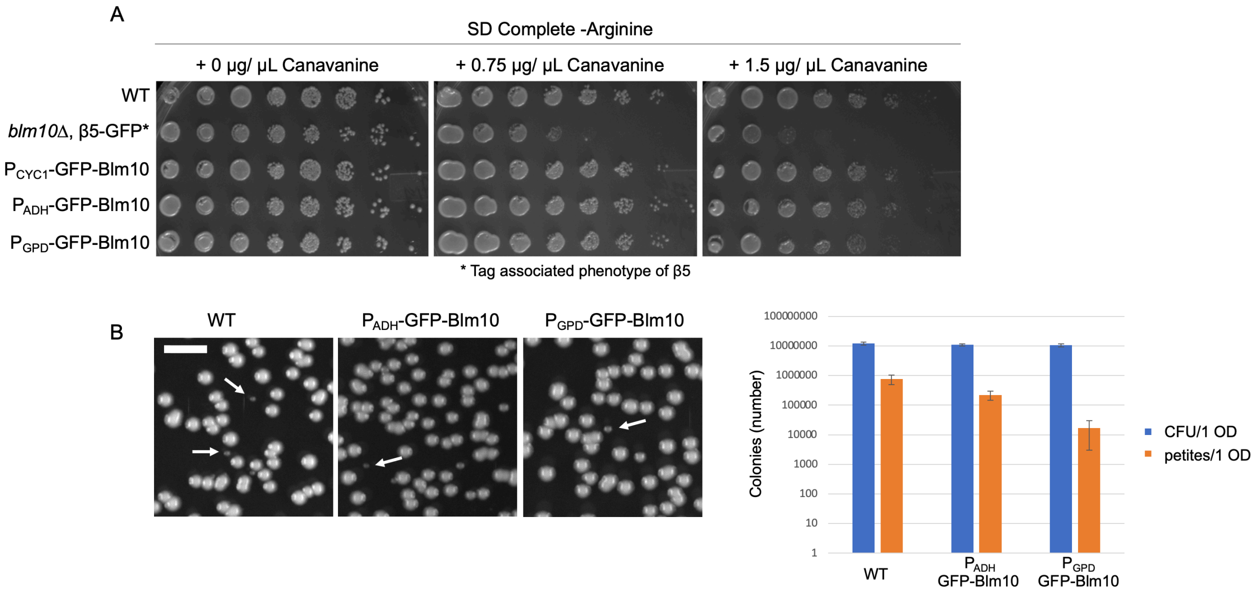

Supplementary Figure 2 - Blm10 overexpression is not associated with growth phenotype on canavanine

(a) A phenotype screen was conducted as in 3C. 1.0 (OD_{600}) cell equivalents were diluted seven-fold and plated on SD complete plates lacking arginine and containing increasing amounts of canavanine (0-1.5 µg/µL). Plates were incubated at 30 degrees for 48 hours. Overexpression of Blm10 was not associated with a growth phenotype. (b) Cells expressing Blm10 from either the endogenous, ADH, or GPD promoter were grown to log phase. 1 OD of cells was collected and resuspended in YPD pre-warmed to 42 °C. This suspension was incubated at 42 °C for 30 minutes. Following this acute heat shock, cells were serially diluted and plated on YPD plates. White arrows indicate petite colonies which were confirmed both microscopically and by plating on glycerol containing media which petites cannot survive on. Colony forming units and petites were counted. Scale bar represents 6 mm. Graph represents average data from 3 biological replicates. Error bars indicate SEM.

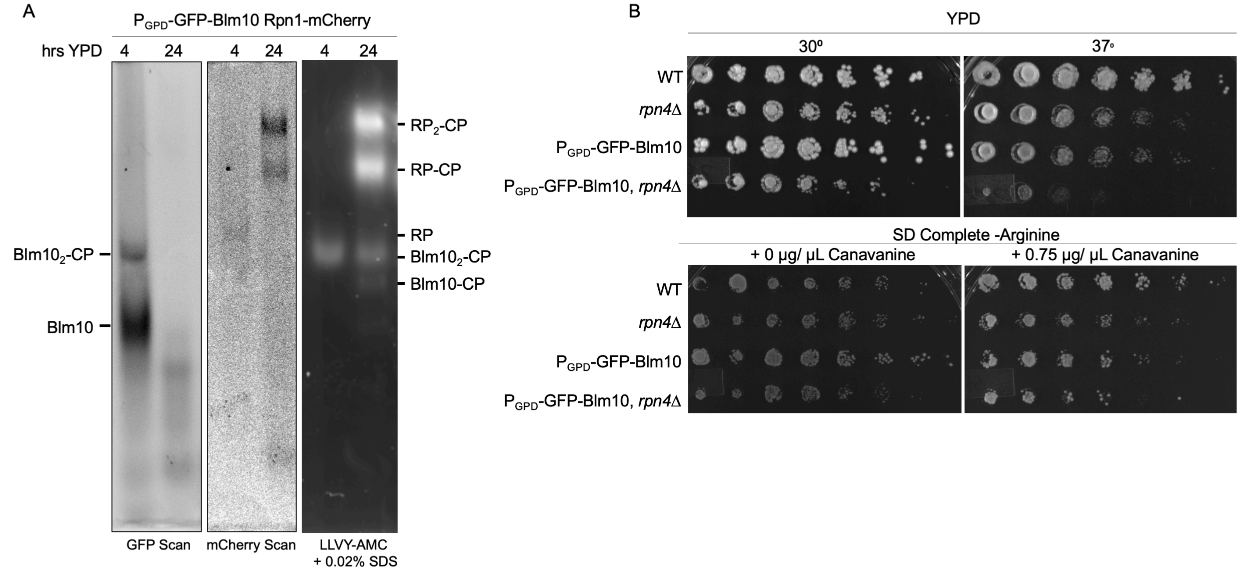

Supplementary Figure 3 - Blm10 overexpression and transcriptional regulation of proteasomes.

(a) Yeast cells expressing Rpn1-mCherry and GPD-GFP-Blm10 were grown to log phase or for 24 hrs. in YPD. 50 ODs of cells were collected and lysed by cryogrinding. Lysates were resolved on native gel and imaged for GFP-Blm10 and Rpn1-mCherry. (b) Indicated yeast strains were grown to log phase in YPD and 1 OD of cells were harvested by centrifugation. 4-fold serial dilutions were carried out and cells were spotted on either YPD plates for growth at 30 °C and 37 °C, or plates lacking arginine (with or without 0.75 µg/mL canavanine) for growth at 30 °C.

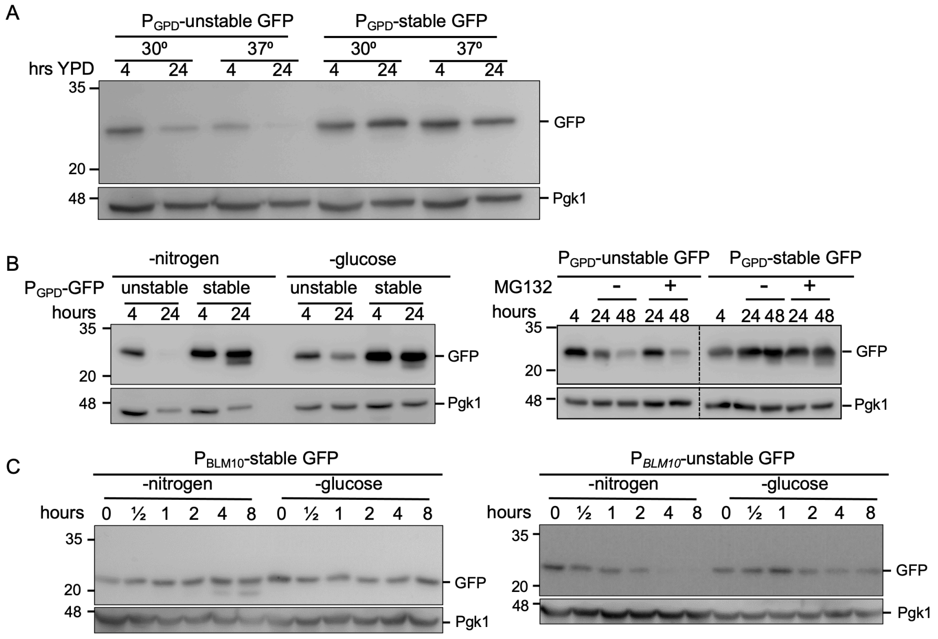

Supplementary Figure 4 – GPD promoter remains active under prolonged cell growth and heat stress

(a) As Blm10 expression decreased during prolonged cell growth, the expression level from the GPD promoter was analyzed using stable and unstable GFP. Constructs were integrated into the URA3-TIM9 region of the genome. Levels of stable GFP remained steady following 24 hours at 30 degrees and 37 degrees. Levels of unstable GFP were reduced at both 30 degrees and 37 degrees, but still detectable indicating the GPD promoter is moderately active. (b) Yeast cells expressing either stable or unstable GFP from a GPD promoter were starved of nitrogen and glucose for 24 hours. 2 ODs of cells were harvested at indicated times and lysed using NaOH lysis. Following electrophoresis, gels were immunoblotted for GFP and Pgk1 (right). Yeast strains from above were grown to log phase, then treated with proteasome inhibitor MG132 (100 μM). 2 ODs were collected at indicated times and lysed using NaOH. GFP and Pgk1 blots were carried out as above. (c) Yeast cells expressing stable or unstable GFP from a BLM10 promoter were grown to log phase then starved of either nitrogen or glucose. 2 ODs of cells were collected and lysed using NaOH lysis method. Lysates were resolved on SDS-PAGE and blotted for GFP and PgK1.

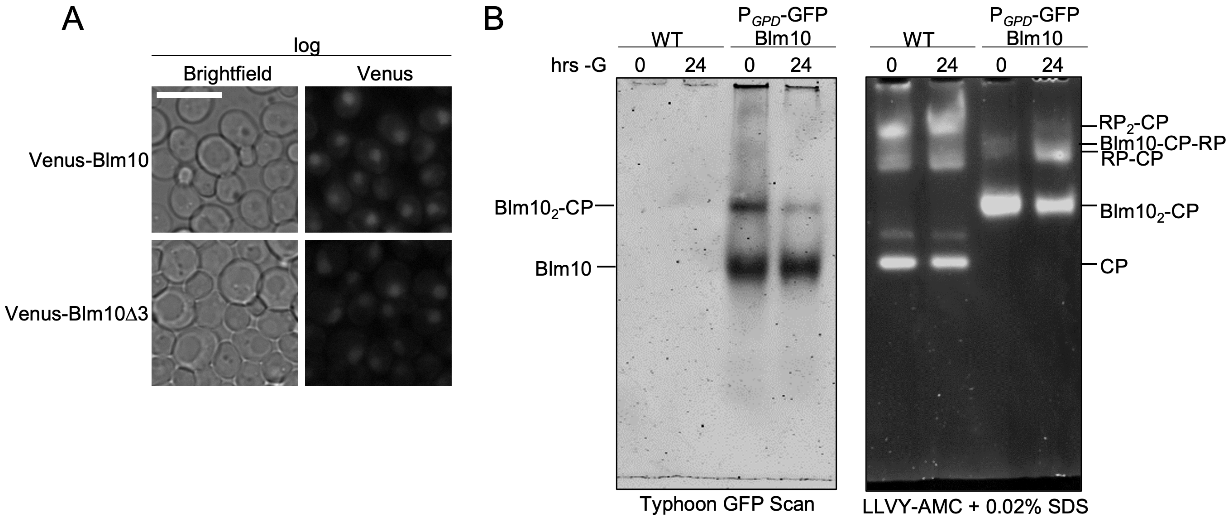

Supplementary Figure 5 - Blm10 levels are not affected by glucose starvation

(a) Strains expressing either bound or unbound Venus-Blm10 were monitored for cellular localization using fluorescent microscopy. Both strains showed nuclear signal indicating both forms of Blm10 can be imported into the nucleus. Scale bar represents 5 µm. (b) Whole cell lysates for endogenous and GPD controlled expression of Blm10 were monitored by native gel electrophoresis following 24 hours of glucose starvation. Following starvation, unbound Blm10 remains present (left panel, lane 4). Despite this observation, RP-CP complexes begin to reform in the absence of glucose indicating that this condition is unfavorable for Blm10-CP complexes (right panel lane 3 versus lane 4).

Supplementary table 1- Strains

Strain	Genotype	Figure
sUB61	*MATα lys2-801 leu2-3, 2-112 ura3-52 his3Δ200 trp1-1*	2A-C, 3B-F,S2A-B,S3B, S5B
Ump1Tap	*MATA ump1::UMP1-CBP-TEV-ZZ-(His3MX6)*	S1A-B
sDL135	*MATα pre1::PRE1-TevProA (HIS3)*	S1A
sJR287	*MATα rpn4::KAN*	3D
sJR288	*MATA rpn4::KAN*	S3B
sJR395	*MATα blm10::CloNAT*	2C,3C-D
sJR858	*MATα pre2::PRE2-GFP (HIS)*	6C
sJR917	*MATα pre2::PRE2-GFP (HIS) blm10::CloNAT*	2B,S2A,6C
sJR966	*MATα blm10::VENUS-BLM10*	2A,2B,5A-C,S5A
sJR989	*MATα blm10::CYC1pGFP-BLM10 (CloNAT)*	2A-2C,3C,S2A
sJR990	*MATα blm10::GPDpGFP-BLM10 (CloNAT)*	2A-2C,3B-F,S2A-B,4A-E,S3B, S5B
sJR991	*MATα blm10::ADHpGFP-BLM10 (CloNAT)*	2A,2B,S2A-B
sJR1001	*MATα blm10::VENUS-BLM10 blm10Y2141-A2143Δ (KanMX)*	5A-C,S5A
sJR1004	*MATα blm10::GPDpGFPBLM10 (CloNAT) atg7::URA*	4A-C
sJR1006	*MATα blm10::VENUS-BLM10 atg7::URA*	5B-C
sJR1007	*MATα blm10::VENUS-BLM10 blm10Y2141-A2143Δ (KanMX) atg7::URA*	5B-C
sJR1012	*MATA ump1::UMP1-CBP-TEV-ZZ-(His3MX6) blm10::GPDpGFPBLM10 (CloNAT)*	S1A
sJR1013	*MATA ump1::UMP1-CBP-TEV-ZZ-(His3MX6) blm10::VENUS-BLM10 blm10Y2141-A2143Δ (KanMX)*	S1B
sJR1014	*MATα blm10::GPDpGFPBLM10 (CloNAT) blm10Y2141-A2143Δ (KanMX)*	2C,3B-D
sJR1016	*MATα blm10::CYC1pGFPBLM10 (CloNAT) blm10Y2141-A2143Δ (KanMX)*	2C,3C
sJR1034	*MATα ura3-1::pGPD-Ubi-Y-eGFP unstable (HIS)*	S4A-B
sJR1035	*MATα ura3-1::pGPD-Ubi-M-eGFP stable (HIS)*	S4A-B
sJR1047	*MATα ura3-1::pBLM10-Ubi-Y-eGFP unstable (HIS)*	3A, S4C
sJR1048	*MATα ura3-1::pBLM10-Ubi-Y-eGFP stable (HIS)*	S4C
sJR1062	*MATα pre1::PRE1-TevProA (HIS3) blm10::GPDpGFPBLM10 (CloNAT)*	S1A
sJR1084	*MATα scl1::SCL1-GFP (HIS)*	1A-1C,6D
sJR1199	*MATα scl1::SCL1-GFP (HIS) blm10::CloNAT*	1B-1C,6D
sJR1202	*MATα blm10::GFP-BLM10 scl1::SCL1-mCherry (KanMX)*	6B,6E
sJR1337	*MATA scl1::SCL1-mCherry (KanMX) blm10::GPDpGFPBLM10 (CloNAT)*	3G,6A
sJR1486 (YMW3)	*MATA his3-11,15 leu2-3,112 ura3-52 can GAL pre2::PRE2-GFPS (HIS3-URA3) HTA2-RFP (natMX) blm10::HIS3*	6C
sJR1487 (YMW1)	*MATA his3-11,15 leu2-3,112 ura3-52 can GAL pre2::PRE2-GFPS (HIS3-URA3) HTA2-RFP (natMX)*	6C
sJR1503	*MATα blm10::GPDpBLM10 (CloNAT) ura3-1:: BLM10pUbi-Y-eGFP unstable (HIS)*	3A

sJR1569	*MATA ump1::UMP1-CBP-TEV-ZZ-(His3MX6) blm10::GPDpGFPBLM10 (CloNAT) blm10Y2141-A2143Δ (KanMX)*	S1C
sJR1743	*MATα blm10::GPDpGFP-BLM10 (CloNAT) rpn1::RPN1-mCherry (KanMX)*	S3A
sJR1757	*MATAα blm10::GPDpGFP-BLM10 (CloNAT) rpn4::(KanMX)*	3F, S3B
MAHQ1	*MATA pdr5*Δ with *uba1-204*	4D-E (ref: [1])
sJR1758	*MATα blm10::GPDpGFP-BLM10 (CloNAT) uba1-204*	4D-E *

** Strain derived from dissection of cross between sJR990 and MAHQ1.*

Supplementary table 2 - Primers

Manipulation	Frwd 5' – 3'	Rvrs 5' – 3'	Template
Venus-Blm10	pRL375<br>GTTAGCTAGCTTTGCACATTAATTTTTCGAT TTGTTACCGcggccgccagggg	pRL376GAATGGGTGATTTGATATCATCGTC ATTGTTAGCGGTCATtttgtacaattcatccataccatggg	pJR 543 (pSH47)
blm10Δ	pf2Blm10<br>CTGTCATCAGGGCTTG	pr2Blm10<br>GTTGATCATTCTCAGTGG	sJR395 gDNA
pre3-GFP	Frwd/Beta5<br>TAT TTT GGA AGG TCA AGG AAG AGG AAG GAT CTT TCA ACA ACG TTA TTG GCC GTA CGC TGC AGG TCG AC	S2- Beta5<br>TAA TGT ATC ATT AAT ATA GAT GTG CAT ATA CAT ATG TTT GAT GCT TCT ATA TCG ATG AAT TCG AGC TCG	pNU293 (pYM28)
P_{CYC1}GFP-Blm10	pRL426<br>GTTAGCTAGCTTTGCACATTAATTTTTCGAT TTGTTACCGCGTACGCTGCAGGTCGAC	pRL427<br>GAATGGGTGATTTGATATCATCGTCATTGT TAGCGGTCATCATCGATGAATTCTCTGTCG	pNU325 (pYM-N13)
P_{ADH}GFP-Blm10	pRL426	pRL427	pNU321 (pYM-N9)
P_{GPD}GFP-Blm10	pRL426	pRL427	pNU329 (pYM-N17)
blm10Δ3	pRL408<br>AGG AAC TGG AAG ACC TGG AGG GTG TCC TAT GGA GAA GTG CCG CCG CCT GAG CGA ATT TCT TAT GA	pRL409<br>GAT GTA CAT ATA TGT CTA GAT ATG TGC TTA ATA TCC TAT ACT AAT ATG AAA TCG ATG AAT TCG AGC TCG	pNU170 pFA6a-GFP (S65T)-kanMX6
atg7Δ	pRL236 -<br>TTC ATT ATA TTT CAA CAA ATA TAA GAT AAT CAA GAA TAA ACG TAC GCT GCA GGT CGA CG	pRL237<br>CGG AAA GTG GCA CCA CAA TAT GTA CCA ATG CTA TTA TAT GCA TCG ATG AAT TCG AGC TCG	pNU166 (pAG60)
P_{GPD}unstable-GFP	pRL495<br>ATGCAGATTTTCGTCAAGACTTTGACCGG	pRL496 GGGTACCGGGTAATAACTG	pJR740 (pNC1124)
P_{GPD}stable-GFP	pRL495<br>ATGCAGATTTTCGTCAAGACTTTGACCGG	pRL496 GGGTACCGGGTAATAACTG	pJR741 (pNC1125)
scl1-GFP	pRL589<br>TGC TGA GAA CAT CGA AGA AAG GCT AGT AGC AAT TGC TGA ACA AGA TCG TAC GCT GCA GGT CGA C	pRL36<br>GTG TTG ACG CGT GTG ATT TCA CAT TAT GTT GTG GCA GGA AGA TCG ATG AAT TCG AGC TCG	pNU293 (pYM28)
scl1-mCherry	pRL600<br>TGCTGAGAACATCGAAGAAAGGCTAGTAGC AATTGCTGAACAAGATggtcgacggatccccggg	pRL36<br>GTG TTG ACG CGT GTG ATT TCA CAT TAT GTT GTG GCA GGA AGA TCG ATG AAT TCG AGC TCG	pJR655- (pBS34)

1. Yu H, Singh Gautam AK, Wilmington SR, Wylie D, Martinez-Fonts K, Kago G, Warburton M, Chavali S, Inobe T, Finkelstein IJ, Babu MM, Matouschek A. **Conserved Sequence Preferences Contribute to Substrate Recognition by the Proteasome**. *J Biol Chem. 2016;291(28):14526-39.* Epub 2016/05/27. doi: 10.1074/jbc.M116.727578. PubMed PMID: 27226608; **PMCID: PMC4938175**.
